# A comprehensive pathological and molecular investigation of viral co-infections in ducks in Egypt

**DOI:** 10.3389/fmicb.2025.1522669

**Published:** 2025-05-08

**Authors:** Rania I. Mohamed, Samah M. Mosad, Hanaa S. Ali, Wejdan Oudah Albalawi, Hanaa A. Elsamadony, Neven M. Ramzy, Alaa S. Saad, Deema Fallatah, Lina Jamil M. Abdel-Hafez, Ashraf Albrakati, Ehab Kotb Elmahallawy

**Affiliations:** ^1^Department of Pathology, Animal Health Research Institute, Mansoura Branch (AHRI), Agricultural Research Center (ARC), Giza, Egypt; ^2^Department of Virology, Faculty of Veterinary Medicine, Mansoura University, Mansoura, Egypt; ^3^Department of Clinical Laboratory Science, Faculty of Applied Medical Sciences, Jouf University, Qurayyat, Saudi Arabia; ^4^Department of Poultry Diseases, Agricultural Research Center (ARC), Animal Health Research Institute (AHRI), Giza, Egypt; ^5^Department of Virology, Agricultural Research Center (ARC), Animal Health Research Institute, Ismailia Branch (AHRI), Giza, Egypt; ^6^Department of Biotechnology, Agricultural Research Center (ARC), Animal Health Research Institute (AHRI), Giza, Egypt; ^7^Department of Medical Laboratory Sciences, College of Applied Medical Sciences, Prince Sattam bin Abdulaziz University, Al-Kharj, Saudi Arabia; ^8^Department of Microbiology and Immunology, Faculty of Pharmacy, October 6 University, Cairo, Egypt; ^9^Department of Human Anatomy, College of Medicine, Taif University, Taif, Saudi Arabia; ^10^Grupo de Investigación en Sanidad Animal y Zoonosis (GISAZ), Departamento de Sanidad Animal, Universidad de Córdoba, Córdoba, Spain; ^11^Department of Zoonoses, Faculty of Veterinary Medicine, Sohag University, Sohag, Egypt

**Keywords:** RT-PCR, duck, NDV, DHAV, AIV, histopathology, phylogenetic, zoonotic

## Abstract

**Introduction:**

Duck production in Egypt plays a significant role in the poultry sector. However, viral infections, such as avian influenza virus (AIV), Newcastle disease virus (NDV), and duck hepatitis A virus (DHAV), pose a significant threat to ducks, leading to substantial economic losses. Despite their impact, data on these duck pathogens in Egypt remain limited.

**Methods:**

In this study, 200 samples from various organs were collected from 20 commercial duck farms and pooled into 20 working samples. Samples of brain, liver, spleen, trachea, and lung were analyzed to detect DHAV, NDV, and H5 and H9 AIV using reverse transcriptase polymerase chain reaction (RT-PCR); then, positive samples were subjected for sequencing. Samples from the same organs were also subjected for histopathological examination.

**Results:**

Interestingly, the RT-PCR detected DHAV, NDV, and H9-AIV, and mixed viral infections were confirmed in some farms. The phylogenetic analysis of DHAV 3D gene revealed that both DHAV-1 and DHAV-3 genotypes are circulating in Egyptian duckling with most tested samples containing DHAV-3 genotype, considered the vaccine used in Egypt contains DHAV-1 strain only. All detected NDV strains in this study are clustered in Genotype VII.1.1 with F0 cleavage site (RRQKR ↓ F) of velogenic NDV. On the other hand, our studied H9-AIV strains are aligned in H9.4.1.1 sub-lineage with other Egyptian field and vaccine seed strains. Local Egyptian vaccine seed strains were found closely related to our isolates than imported vaccines. H9.4.1 strains displayed HA0 protein cleavage site motif PARSSR^↓^GLF of LPAI. All the aligned Egyptian H9-AIV field and local vaccine strains have 168 N, 191H, 197 T, 224 L, and 234 L amino residues, indicating that these viruses had the characteristic of receptor specificity like that of human influenza virus increasing the zoonotic risk of such virus. Histopathologically, animals showed characteristic lesions in various organs coherent to the infection by these mentioned pathogens.

**Conclusion:**

Collectively, the study provided novel information about viral infections linked to neurological diseases of ducks in Egypt and concluded that local DHAV vaccine needs to be modified to contain both DHAV-1 and DHAV-3 strains.

## 1 Introduction

Duck production is regarded as the second most significant poultry species production worldwide following chickens. It is therefore not surprising to mention that duck farming has attracted a lot of attention worldwide due to its increased productivity in contrast to other bird species (El-Soukkary et al., 2005). In Egypt, there were approximately 5.63 million ducks reared in 2019 which play a significant role in poultry industry (Galal, 2022). Compared to chickens, domestic ducks exhibit greater adaptability to varying environmental and rearing conditions, lower maintenance requirements, a higher feed conversion ratio, quick growth, and a relative resistance to common avian diseases (Adzitey and Adzitey, 2011). Despite ducks are significantly less prone to illness than chickens due to inherent genetic resistance, they are susceptible to numerous bacterial, viral, and parasitic infection in addition to malnutrition and mycotoxicosis (Abd, 2021). Viral diseases such as duck hepatitis A virus (DHAV), Newcastle disease virus (NDV) and avian influenza virus (AIV) pose the most significant risks to duck production, among others (Patil et al., 2021). Duck viral hepatitis (DVH) is considered an emerging notifiable virus that affects duckling which causes significant financial losses (WOAH, 2023). The Egyptian duck industry is badly impacted by these losses, which also include low growth rates, mortality, and increased preventive and control expenses (Abd, 2021). Three types of DVH (I, II, and III) are recorded; type I DVH is caused by three distinct genotypes (El-Soukkary et al., 2005; Galal, 2022; Adzitey and Adzitey, 2011) of the duck hepatitis A virus (DHAV). DHAV-1, 2, and 3 are classified in the *Picornaviridae* family (genus *Avihepatovirus*) with DHAV-1 being the most pathogenic genotype. Meanwhile, type II DVH is caused by duck astrovirus type 1, primarily in the United Kingdom (UK), causing similar pathological changes to that of type I DHV. Finally, type III DVH is caused by duck astrovirus type 2, a distinct less virulent virus, causing similar liver lesions in young ducklings in United States of America (WOAH, 2023). Newcastle disease (ND), which is caused by NDV, is a highly contagious, infectious, and fatal disease affecting different avian species (Zahid et al., 2022). NDV is classified as *Orthoavulavirus javaense*, which classified in the genus *Orthoavulavirus* of subfamily *Avulavirinae* and family *Paramyxoviridae* (ICTV, 2023). Previously known as NDV, *Orthoavulavirus javaense* can infect over 200 bird species. The severity of the disease varies according the host and virus strain, and even low virulence strains can cause severe respiratory and digestive diseases when worsened by other infectious agents or environmental factors (WOAH, 2021). In this regards, chickens and turkeys are the most vulnerable species, while ducks and geese are the least susceptible and thought to be NDV reservoirs (Zahid et al., 2022; Miller and Koch, 2013). Many strains of NDV, exhibiting varying degrees of virulence, have been identified in both sick and clinically healthy ducks (Zhang et al., 2011). The number of ND cases in ducks has been steadily rising in recent years, suggesting that, in addition to serving as carriers and reservoirs of NDV, ducks may also be susceptible to certain virus strains (Xu et al., 2017; Elbestawy et al., 2019). Influenza infections in bird species are caused by viruses from the *Orthomyxoviridae* family, *Alphainfluenzavirus* genus, and *Alphainfluenzavirus influenzae* species (formerly known as influenza A virus) (ICTV, 2023). Influenza A virus subtyping is dependent on neuraminidase (N) and hemagglutinin (H) antigens. Currently, there are 16 H subtypes (H1–H16) and 9 N subtypes (N1–N9) of influenza A, with the possibility of additional bat originated subtypes (H17N10 and H18N11) (WOAH, 2025; Wu et al., 2014). Highly pathogenic avian influenza (HPAI) viruses are primarily associated with the H5 and H7 subtypes. While H5 AIVs are classified as HPAI, low pathogenic variants of these subtypes are commonly found in aquatic wild birds and poultry. It should be noted that HPAI worldwide spread has increased, and H5 or H7 low pathogenic viruses may become highly pathogenic by mutation (WOAH, 2025). It has also been shown that a diverse range of bird species can be infected by the influenza A virus, with ducks and other waterfowl considered natural reservoirs for all influenza subtype (WOAH, 2025; Marchenko et al., 2012). Domestic ducks infected with H5N1 viruses can develop subclinical, clinical, or asymptomatic illnesses, and the asymptomatic ducks often excrete the virus through respiratory droplets and feces (Hulse-Post et al., 2005; Sturm-Ramirez et al., 2005).

Ducks raised under various conditions, including backyard, free-range, and nomadic systems, are considered vital to the persistence and dissemination of the virus among both commercial and wild bird populations (Bi et al., 2016; Hassan et al., 2020; Jiang et al., 2017). However, HPAI H5N1 virus strains from Asia have demonstrated a wide range of harmful effects to domestic ducks, ranging from total lack of clinical symptoms to severe neurological impairment and death. Infections caused by low pathogenic avian influenza (LPAI) viruses typically affect the digestive and respiratory systems of birds. Notably, shedding of influenza viruses from both the respiratory and intestinal tracts has been documented in ducks, occurring in cases of both symptomatic and asymptomatic infections (Truszczyński and Samorek-Salamonowicz, 2008). Although AIV H9N2 is classified as a low pathogenic strain, mixed infections can lead to significant illness and high mortality rates in affected birds (Seifi et al., 2010). The pathogenicity of AIV H9N2 varies and is influenced by the presence of co-infections. Globally, AIV H9N2 is widely disseminated among domestic poultry (Karimi-Madab et al., 2010). Given the endemic nature of HPAI H5N1 and the presence of other infections in conditions of inadequate biosecurity, the emergence of H9N2 in backyard ducks poses an additional risk to Egypt’s poultry industry (Fiala et al., 2018).

Over the past decade, there has been an increase in mixed co-infections among poultry, particularly in developing countries and intensive farming systems. Outbreaks of co-infections with high mortality rates and diverse clinical manifestations have become more frequent in commercial chicken flocks in Egypt (Mansour et al., 2021). Furthermore, mixed infections involving AIV, NDV, infectious bronchitis virus (IBV), and infectious laryngotracheitis virus (ILTV) have been reported in both domestic and wild birds in Egypt (Shehata et al., 2019). Notably, a co-infection involving three subtypes of AIV (H5N1, H9N2, and H5N8) was also detected (Hassan et al., 2021). Mixed infections of NDV and AIV have been also identified in pigeons and various species of migratory wild birds (Mansour et al., 2017; Sultan et al., 2022). In addition, in ducks, co-infections of highly pathogenic AIV and DHAV have been observed in Egypt (Mansour et al., 2018). Previous literature on viral diseases in ducks from Egypt indicates a paucity of comprehensive studies examining neurologic diseases linked to viral infections using pathological and molecular methods. Therefore, this study aims to evaluate the pathological and molecular aspects of a complex of viral infections in ducks that induce neurologic symptoms, specifically focusing on DVAH, NDV, and AIV.

## 2 Materials and methods

### 2.1 Ethical statement

This study was approved by the Mansoura University Animal Care and Use Committee (MU-ACUC), Egypt, under approval code number VM.R.24.01.140.

### 2.2 Sample collection and preparation

Clinical samples (*n* = 200) were collected from 20 duck flocks (Pekin, Mullard, and Baladi) ranging in age from 1 day to 3 months, all exhibiting nervous symptoms and varying mortality rates (2–93%) across the Dakahlia, Gharbia, and Damietta Governorates in Egypt. From each farm, 10 sick birds (either freshly deceased or moribund) were necropsied, and their organs were collected for analysis. For molecular studies, parts from the brain, liver, spleen, trachea, and lung were combined and treated as a single sample. In addition, a negative control sample was prepared by pooling the same organs from 10 euthanized healthy ducklings, each 10 days old. The collected samples (pooled brain, liver, spleen, trachea, and lung) were homogenized with phosphate-buffered saline (PBS) and then centrifuged at 3000 rpm for 15 min. The resulting supernatant was stored at −20°C until use for the molecular detection of the referred pathogens (Woolcok, 1998). In addition, subsequent samples, including the other parts from brain, liver, spleen, trachea, and lung, were collected from the necropsied ducks for histological analysis. Comprehensive details of the analyzed samples, including sample ID, bird age, breed, mortality rate, RT-PCR results for each virus, accession numbers, and genotypes of sequenced samples, are presented in Supplementary Table S1.

### 2.3 Molecular characterization of DHAV, NDV, and AIV

#### 2.3.1 Extraction of viral RNA

QIAamp^®^ MinElute^®^ Virus Spin Kit (QIAGEN, GmbH, Germany, Cat. No. 57704) was used for extraction of viral RNA from obtained samples. In brief, tissue homogenates (200 μL) were added to Buffer AL (200 μL), and the mix was incubated for 15 min at 56°C. Approximately 250 μL of pure ethanol (96%) was added to the mix and then incubated at room temperature (25°C) for 5 min. After transferring the solutions to QIAamp MinElute columns and centrifuging them for 1 min at 8000 rpm, the viral RNA was washed twice with 500 μL of AW1 buffer and 500 μL of AW2 buffer. The viral RNAs were then eluted from each column using 40 μL of AVE buffer. The eluted RNAs were collected and stored at −80°C until used for RT-PCR.

#### 2.3.2 Reverse transcriptase polymerase chain reaction

RT-PCR was employed for the molecular confirmation of DHAV, NDV, H5-AIV, and H9-AIV in the collected samples through the partial amplification of the 3D, F, H5, and H9 genes, respectively, using primers that had been previously designed and illustrated in Table 1 (OIE Terrestrial Manual, 2023; Kant et al., 1997; Akhter et al., 2016; Hussein et al., 2014). These primers were purchased from Metabion International AG, Germany, and the Verso 1-Step RT-PCR Reddy Mix Kit (Thermo Fisher Scientific, Inc., United States, Cat. No: AB1454LDB) was utilized for RT-PCR. To obtain a 50 μL reaction, the reaction mixture was composed of 1 μL Verso Enzyme Mix, 25 μL 1-Step PCR Reddy Mix (2X), 2.5 μL RT Enhancer, 50 ng extracted RNA, 1 μL (10 μm) of each primer, and water (free of DNase/RNase) to 50 μL. Adjusting the T gradient Biometra thermal cycler (Germany) resulted in one cycle of two steps: cDNA synthesis (50°C/15 min) and Verso inactivation (95°C/2 min). After that, there were 35 cycles consisting of three steps: denaturation (95°C/20 s), annealing (52°C for DHAV, 50°C for NDV and H9 AIV, and 57°C for H5 AIV) for 1 min then extension (1 min at 72°C) after which comes one final extension cycle (72°C/5 min). The resulting PCR products were electrophoresed against a 100 bp DNA ladder (Jena Bioscience, Germany) in an agarose gel at a concentration of 1.5% in 0.5X Tris-Borate-EDTA (TBE) buffer. Next, the acquired DNA bands were examined using a UV transilluminator (Vögtlin et al., 1999). Using a sterile scalpel, bands of the appropriate sizes from the selected PCR-positive samples (sharp bands) were excised from the gel and placed in 1.5 mL Eppendorf tubes containing TBE buffer. DNA extraction from these excised agarose gel was performed using an Agarose Extraction Kit (QIAGEN, GmbH, Germany) following the manufacturer’s instructions, as described by Lee et al. (2012).

**Table 1:** Primers used for partial amplification of specific genes in DHV, NDV, H5 AIV, and H9 AIV.

Primer	Sequence (5′–3′)	Virus	Gene	Amplicon length (bp)	References
DHAV F	5′-AAGAAGGAGAAAATYAAGGAAGG-3′	DHAV	3D	467	OIE Terrestrial Manual (2023)
DHAV R	5′-TTGATGTCATAGCCCAASACAGC-3′				
NDV F	5′-TTG ATG GCA GGC CTC TTG C-3′	NDV	F	248	Kant et al. (1997)
NDV R	5′-AGC GTY TCT GTC TCC T-3′				
H9 F	5′-AGCAAAAGCAGGGGAACTCC-3′	H9 AIV	H9	808	Akhter et al. (2016)
H9 R	5′-CCATACCATGGGGCAATTAG-3′				
H5 F	5-′ GATTGTAGTGTAGCYGGATGG-3′	H5 AIV	H5	406	Hussein et al. (2014)
H5 R	5-′CTTGTCTGCTCTKCNKCATC-3′				

#### 2.3.3 DNA sequencing and sequence analysis

Using the same primer sets used in RT-PCR, gel extracted DNAs from chosen samples were sent to MACROGEN laboratory, Korea, for two-directional DNA sequencing of the DHAV (3D gene), NDV (F gene), and H9 AIV (H9 gene). The sequenced genes’ obtained nucleotide sequences were then deposited in GenBank,^1^ along with the accession numbers shown in Supplementary Table S1. The DNA sequences that were acquired were compared to other sequences in GenBank using MEGA X software (Kumar et al., 2018). Using the 1,000 Bootstrap repeats test and the kimura-2-parameter model, neighbor-joining distance trees were constructed for DHAV-3D gene and H9 gene of H9-AIV. Maximum likelihood phylogenetic tree was constructed for NDV F-gene with Kimura 2-parameter model and 1,000 bootstrap replications. MEGA X software measured the distance between the samples under study and other strains from GenBank. The viruses under investigation’s obtained nucleotide and deduced amino acid sequences, together with additional sequences from GenBank, were aligned using the Clustal W alignment function in Bioedit software (Hall, 1999).

### 2.4 Histopathological examination

During necropsy of the ducks, tissue samples were collected from the brain, liver, spleen, trachea, and lungs and preserved in 10% neutral formalin. These samples underwent standard histological processing, including paraffin embedding. Thin sections (5 μm) were then prepared, mounted on microscope slides, and stained with hematoxylin and eosin for microscopic examination (Bancroft and Gamble, 2008).

## 3 Results

### 3.1 Clinical symptoms and gross lesions

**Figure 1:**
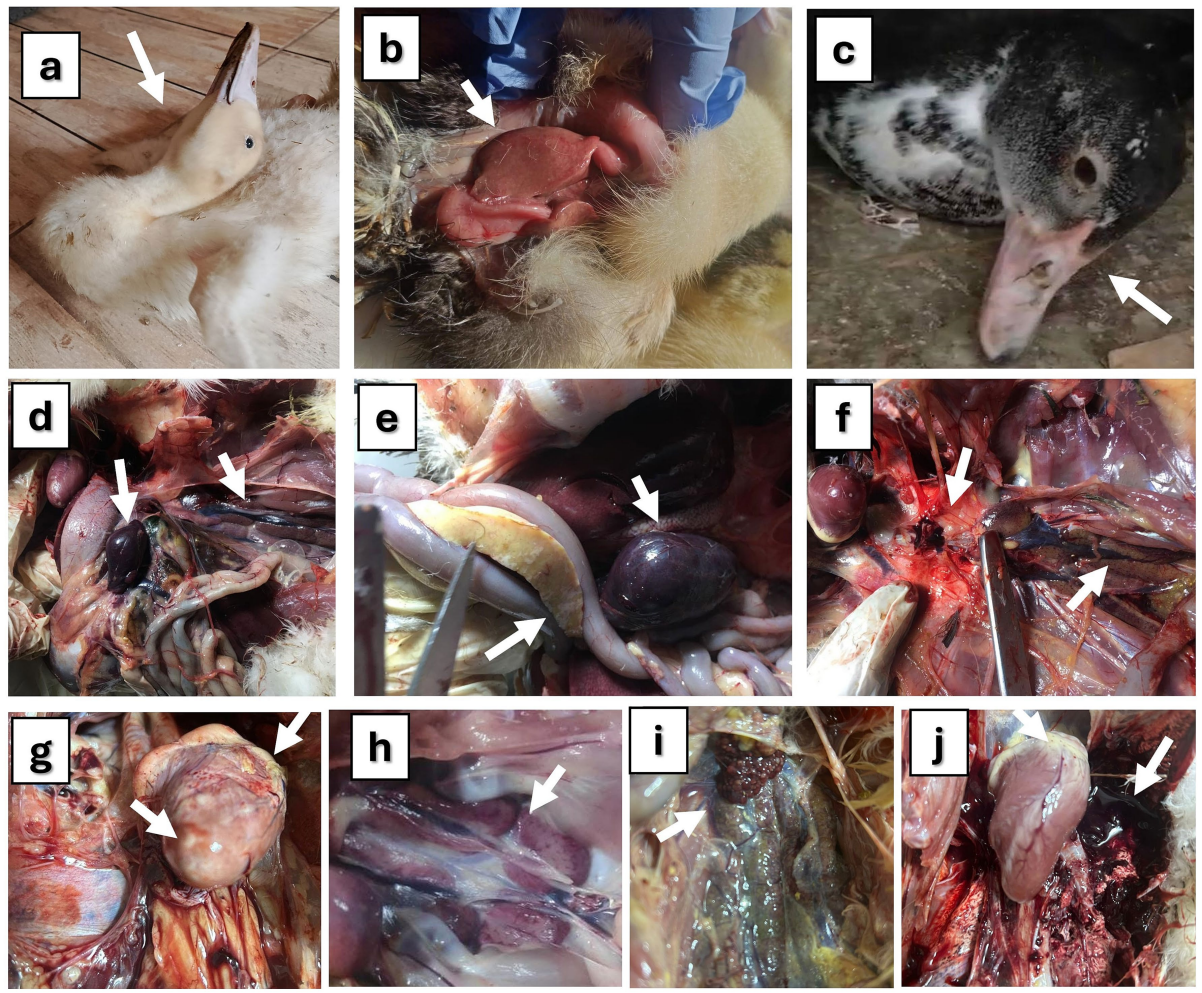
Clinical signs and PM lesions of studied ducks. **(a)** Pekin duck (16 days old) suffering from DVH showing nervous signs (opisthotonus, and torticollis). **(b)** Baladi duckling (10 days old) suffering from duck virus hepatitis showing pale and swollen liver. **(c)** Baladi duck (3 months old) suffering from Newcastle disease showing recumbency and nervous signs on head (shaking). **(d)** Baladi duck (3 months old) suffering from Newcastle disease showing enlarged and congested spleen mottled with whitish foci on the surface (arrow) and nephrosis in kidney (arrow head). **(e)** Pekin duck (3 months old) suffering from avian influenza showing enlarged pancreas (arrow) containing grayish patches together with congested liver and spleen (arrow head). **(f)** Mallard duck (3 months old) suffering from avian influenza showing severe hemorrhagic lungs (arrow) with enlarged kidney lobules (arrow head). **(g)** DHAV/H9-AIV mixed infection in Baladi duck (15 days old), showing necrosis in heart muscles and petechiae on the heart. **(h)** DHAV/H9-AIV mixed infection in Baladi duck (15 days old), showing nephritis with urates deposition. **(i)** DHAV/NDV mixed infection (14 days old) showing severe nephritis. **(j)**: NDV/ H9-AIV mixed infection in Baladi duck (60 days old) showing severe hemorrhage in lung with some petechiae on heart.

The reported clinical signs, PM lesions, and mortality rates varied among the studied flocks, depending on the specific viral infections present on each farm. Ducklings infected with DHAV exhibited prominent clinical signs, including depression, anorexia, fever, emaciation, and feather discoloration. Affected birds also showed watery, greenish-white diarrhea, lethargy, drooping wings, ataxia, and loss of balance. In severe cases, some ducklings were unable to stand, lay on their sides with spasmodic paddling movements, and displayed neurological signs such as opisthotonus and torticollis with 55–92% mortality (Figure 1a). The liver exhibited the most prominent gross pathological changes, appearing pale and enlarged with areas of necrosis (Figure 1b). The spleen and kidneys showed enlargement and swelling. In NDV-infected ducklings, the predominant clinical signs were anorexia, paresis, emaciation, greenish-white diarrhoea, inability to rise, lay on their sides and exhibited a swimming motion with both legs, along with twisting of head and neck, lack of muscular coordination, circling, muscular tremors and torticollis (Figure 1c), with low mortality rate (2–5%). Upon PM examination, all NDV-infected birds had greenish intestinal contents, septicemia, and congestion in their internal organs. In addition, hemorrhages were observed in the proventriculus glands, mucosa, and cecal tonsils. Enlarged and congested spleen with white foci was scattered throughout (Figure 1d). The main clinical symptoms of AIV-infected birds included depression, decreased feed consumption and respiratory signs, sneezing and coughing, rales, nasal discharge, and white-greenish diarrhea with 15–20% mortality. The results of the necropsy showed pancreatic hypertrophy, liver and spleen congestion (Figure 1e), enlarged renal lobules, and dispersed hemorrhagic patches in the lung (Figure 1f). Hepatization and severe congestion of the lung were also observed. In mixed infected flocks, the mortality rate increased with more severe signs and PM lesions. In cases of DHAV and H9-AIV co-infection, the kidney and heart were severely affected, exhibiting cardiac muscle necrosis with petechiae on the heart and severe nephritis with urate deposition (Figures 1g,h). Kidney was worse affected in mixed DHAV with NDV (Figure 1i), while gizzard, intestine, trachea, and lung were severely affected in NDV/H9-AIV mixed infection (Figure 1j). Single viral infection caused lower mortality rates than mixed viral infection. In this context, single infections with DHAV, NDV, or H9-AIV resulted in mortality rates of 55–92, 2–5, and 15–20%, respectively. In contrast, mixed viral infections led to higher mortality rates: DHAV/NDV, DHAV/H9-AIV, and NDV/H9-AIV mixed infections exhibited mortality rates of 70–93, 85–88, and 40–70%, respectively.

### 3.2 Molecular characterization of DHAV, NDV, and AIV

Partial amplification of the DHAV-3D, NDV-F, AIV- H9, and AIV-H5 genes was performed among the 20 examined samples from 20 different farms, using RT-PCR. Where RT-PCR was used to identify both single and mixed viral infections in the farms under study, six farms were found to be exclusively infected with DHAV (farm nos. 3, 9, 10, 11, 16, and 20). Only four farms had NDV infection confirmed (farm nos. 2, 8, 17, and 19), while two more farms had H9-AIV detection (farm nos. 6 and 12). It is interesting to note that mixed viral infections were verified in 8 farms: 2 farms had mixed infections of DHAV and NDV (farm nos. 1 and 14), 2 farms had infections of DHAV and H9-AIV (farm nos. 7 and 15), and 4 farms had infections of both NDV and H9-AIV (farm nos. 4, 5, 13, and 18). Regarding H5-AIV, all farms were tested negative (Supplementary Table S1). All tested samples were confirmed to be free of bacterial infections before virus identification, including *Staphylococcus* species and *Riemerella anatipestifer* that were excluded through PCR testing of all samples.

### 3.3 DNA sequencing and sequence analysis

The sequencing results revealed the presence of both single and mixed viral infections. The sequences of strains OQ376758 and OQ376759 correspond to individual DHAV-3 infections, OQ376754 to NDV, and OQ346185 to H9-AIV. In addition, mixed infections were identified, including DHAV-1/NDV (OQ376755 DHAV-1/OQ376751 NDV), DHAV-3/H9-AIV (OQ376757 DHAV-3/OQ346186 H9-AIV), and NDV/H9-AIV (OQ376752 NDV/OQ346183 H9-AIV and OQ376753 NDV/OQ346184 H9-AIV).

#### 3.3.1 DHAV sequence analysis

**Figure 2:**
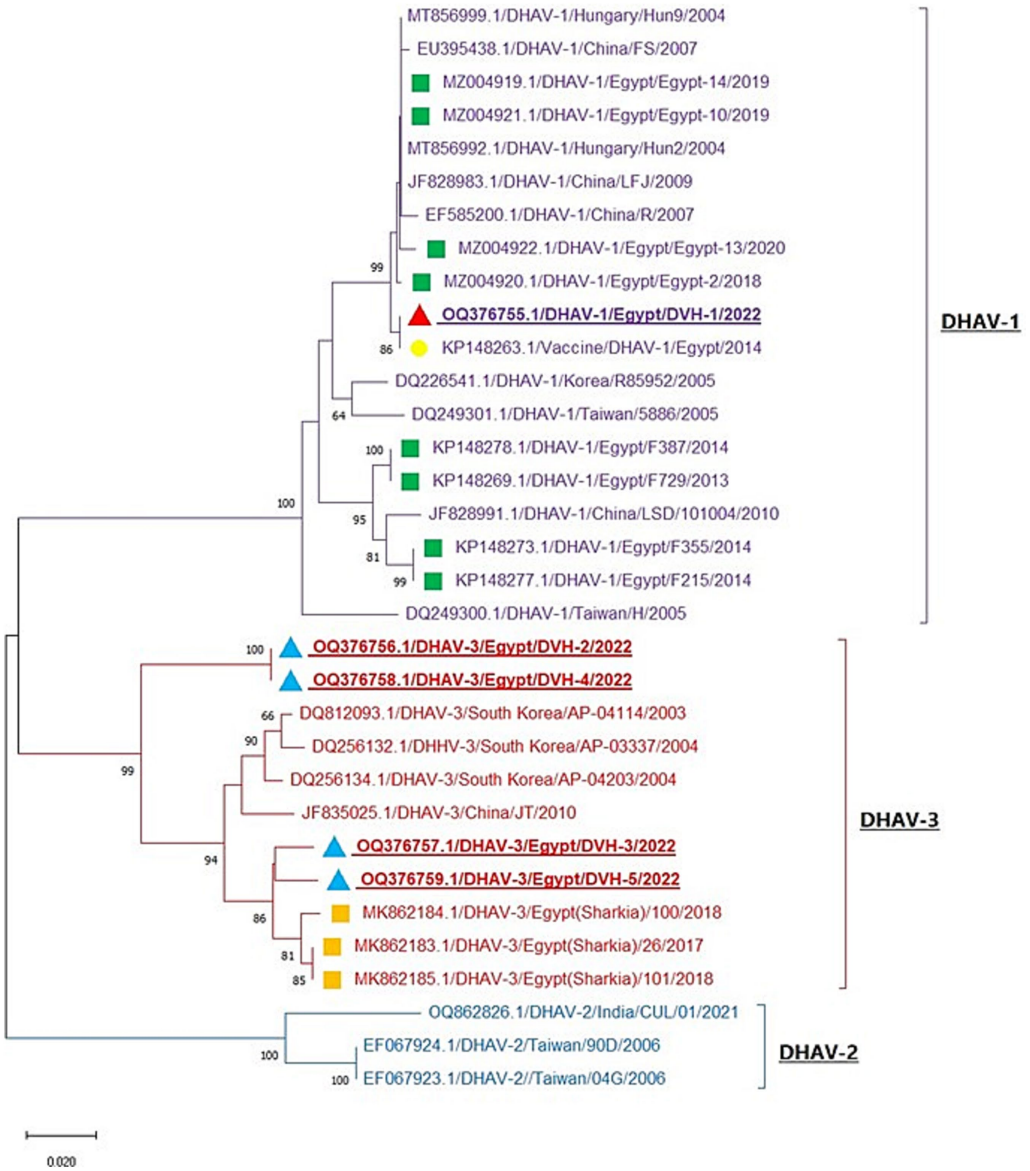
Neighbor-joining phylogenetic tree of DHAV is based on the 3D gene nucleotide sequences analysis. The tree is divided into three clades (DHAV-1, DHAV-2, and DHAV-3). Red and blue triangles: our studied strains; green and orange squares; reference Egyptian isolates from GenBank; yellow circle: Egyptian vaccine strain. The strains OQ376756, OQ376758, and OQ376759 represent individual DHAV-3 infection, while the strain OQ376755 represents DHAV-1/NDV mixed infection and the strain OQ376757 represents DHAV-3/H9-AIV mixed infection.

Phylogenetic analysis of DHAV obtained nucleotide sequences with other sequences from GenBank revealed that the DHAV phylogenetic tree was divided into three genotypes (DHAV-1, DHAV-2, and DHAV-3). One of our detected DHAV strains (OQ376755.1/DVH-1) was aligned in DHAV-1 genotype with other Egyptian strains with 95.1–99.5% homology and was identical to the Egyptian DHAV-1 vaccine strain (KP148263.1) with 100% homology. Interestingly, the other four strains (OQ376756, OQ376757, OQ376758, and OQ376759) are aligned in DHAV-3 genotype with other Egyptian DHAV-3 strains with 93–97.9% identity (Figure 2 and Supplementary Table S2). Our DHAV-3 strains showed 80.3–81.4% identity of vaccine strain used in Egypt. DHAV-2 strains exhibit significant differences from DHAV-1 and DHAV-3, with 79.0–79.9% identity with DHAV-1 strains and 79.6–80.5% identity with DHAV-3 strains (Supplementary Table S2). Using BioEdit software analysis of obtained deduced amino acid sequences with other Egyptian strains, we noticed conserved amino acid substitutions among DHAV genotypes which can be used as amino acid signature for differentiation between DHAV genotypes; these amino acids are illustrated in Table 2 and Supplementary Figure S1. Our DHAV-3 strains (DVH-3 and DVH-5) showed the same DHAV-3 amino acid signature, while the other DHAV-3 strains (DVH-2 and DVH-4) have four amino acid substitutions (F65S, A78E, Q93K, and I108L) when compared to other DHAV-3 reference strains.

**Table 2:** Amino acid substitutions of 3D gene among DHAV genotypes.

Genotype	aa no					
	3	5	15	26	53	54
DHAV-1	D	F	I	C	T	Q
DHAV-2	E	Y	L	A	A	Q
DHAV-3	E	Y	L	S	A	R

#### 3.3.2 NDV sequence analysis

**Figure 3:**
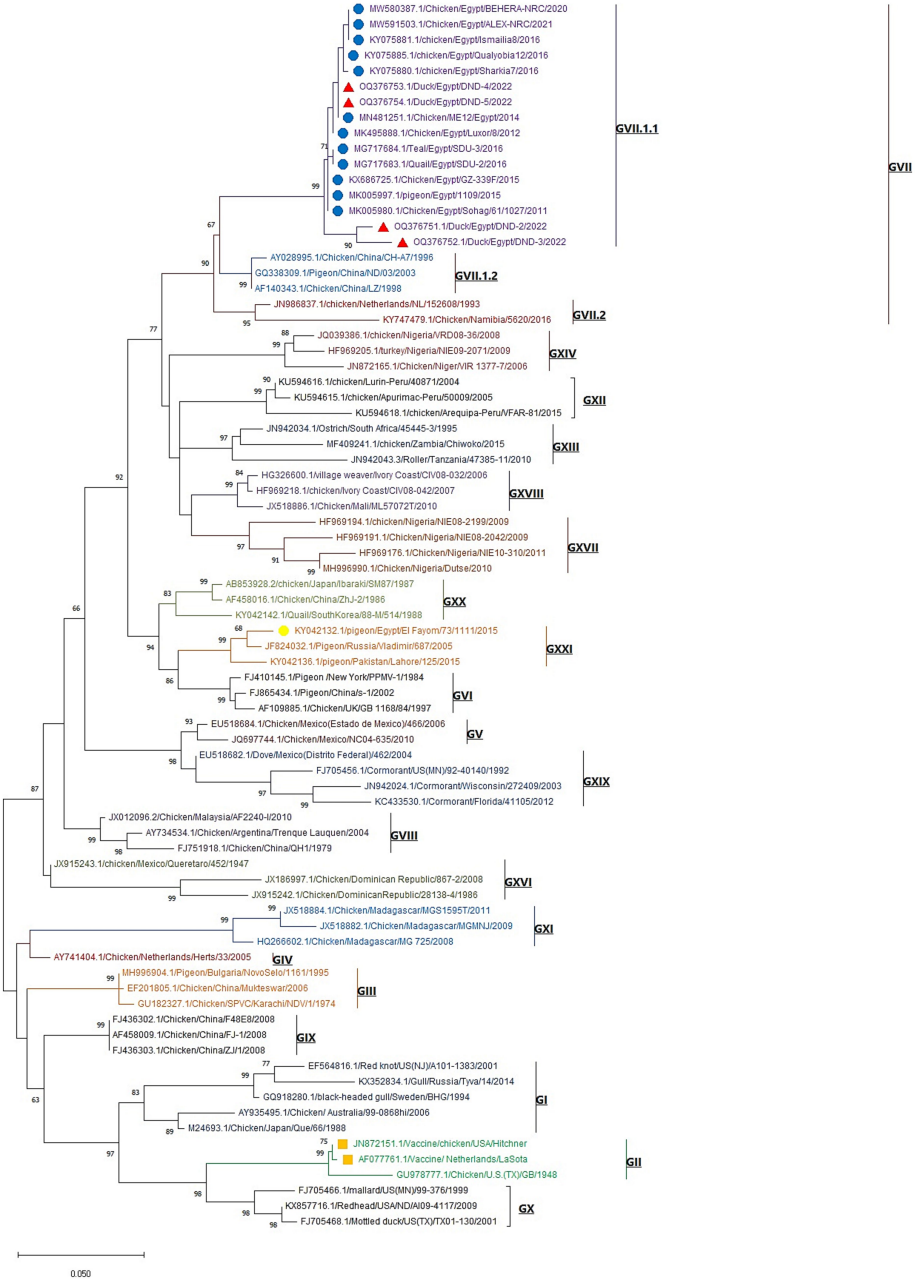
Maximum likelihood phylogenetic tree of NDV F-gene nucleotide sequences representatives of the 20 NDV genotypes. The tree was constructed with Kimura 2-parameter model and 1,000 bootstrap replications. Our studied NDV strains are labeled with red triangles, and other GVII.1.1. Egyptian strains (blue circles), Egyptian GXXI strain (yellow circle), and GII vaccine strains (orange squares) are included in the tree. The strain OQ376754 represents individual NDV infection, while the strains OQ376752 and OQ376753 represent NDV/H9-AIV mixed infection and the strain OQ376751 represents DHAV-1/NDV mixed infection.

The phylogenetic analysis of the obtained Newcastle disease virus (NDV) F gene nucleotide sequences, in conjunction with other sequences from GenBank, revealed that the phylogenetic tree is divided into 20 NDV genotypes (GI-GXXI), with genotype GXV excluded from the analysis due to its inclusion of recombinant NDV strains, as reported by Dimitrov et al. (2019). All detected NDV strains in this study were clustered in Genotype VII.1.1 with other Egyptian Genotype VII.1.1 NDV strains from different host species including chicken, tail, quail, and pigeon with one reference Egyptian strain (El_Fayom/73/1111) aligned in GXXI, whereas vaccine strains are clustered in Genotype II (Figure 3). BioEdit software analysis of deduced amino acid sequences revealed 16 amino acid substitutions in Egyptian GVII.1.1 strains when compared to GII strains (V52I, M69L, R78K, E82D, K101R, G104E, S107T, R112G, K115G, F117L, V121I, S124G, S176A, N192K, R195Q, and T203A). While comparing the amino acid sequences of the F0 cleavage site at positions 112–117 (aa 112–116 at the C-terminus of the F2 protein and the residue 117 of the N-terminus of the F1 protein), we noticed RRQKR^↓^F in all GVII.1.1 strains, KRQKR^↓^F in GXXI Egyptian strain, and GRQGR^↓^L in GII vaccine strains as illustrated in Supplementary Figure S2 and Table 3.

**Table 3:** Amino acid substitutions among deduced amino acids of F protein of NDV genotype GVII.1.1 and GII vaccine strains.

Genotype	aa no.													
	52	69	78	82	101	104	107	112–117	121	124	176	192	195	203
GVII.1.1	V	M	R	E	K	G	S	RRQKRF	V	S	S	N	R	T
GII	I	L	K	D	R	E	T	GRQGRL	I	G	A	K	Q	A

#### 3.3.3 H9-AIV sequence analysis

**Figure 4:**
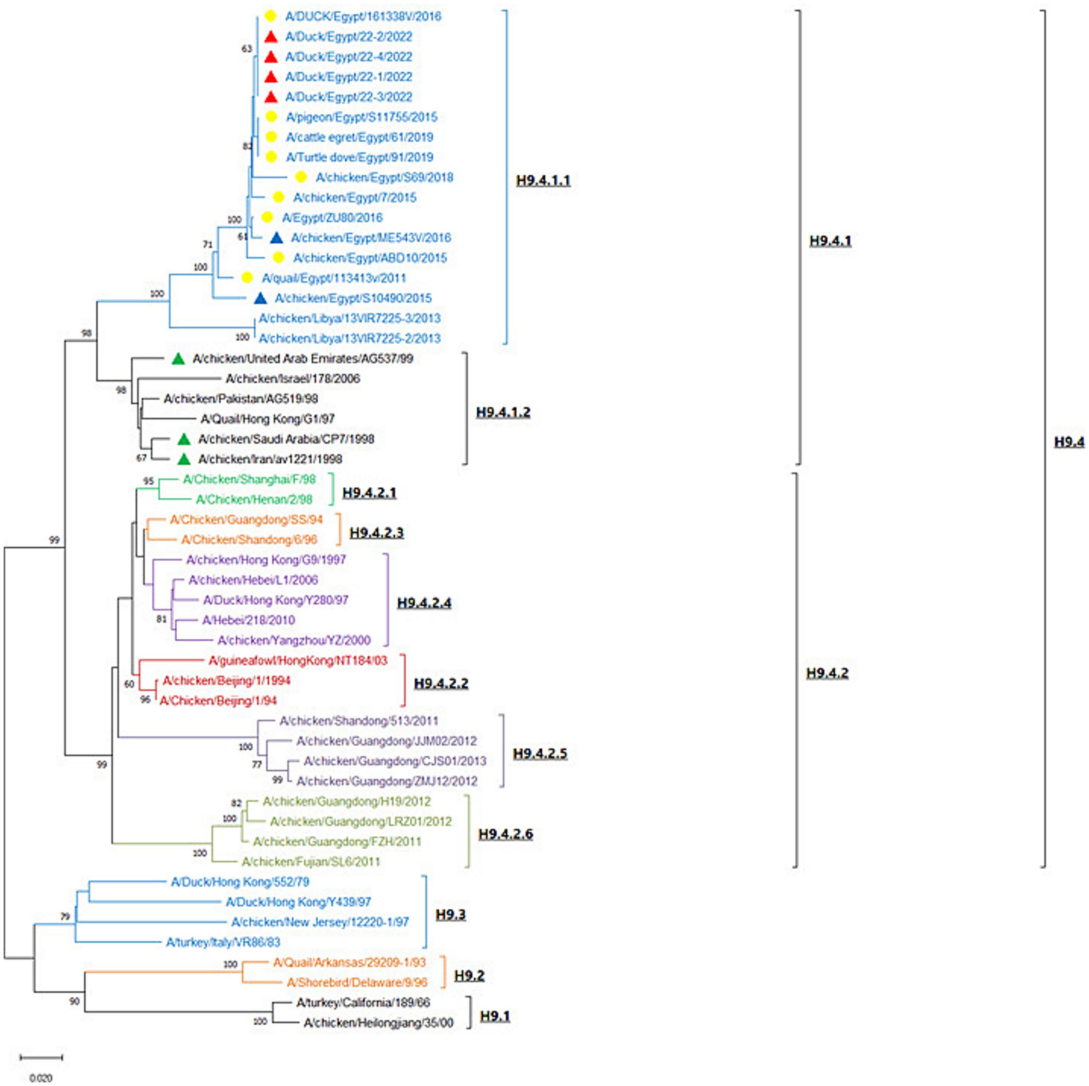
Neighbor-joining phylogenetic tree of H9-AIV which is based on the HA gene nucleotide sequences analysis. The tree was constructed with Kimura 2-parameter model and 1,000 bootstrap replications. The tree is divided into four lineages (H9.1–H9.4). Red triangles represent our studied strains; yellow circles represent reference Egyptian isolates from GenBank; blue triangles represent the local Egyptian vaccine seed strains; green triangles represent the imported vaccine seed strains used in Egypt. The strain OQ346185 represents individual H9-AIV infection, while the strains OQ346183 and OQ346184 represent NDV/H9-AIV mixed infection and the strain OQ346186 represents DHAV-3/H9-AIV mixed infection.

H9-AIV HA gene nucleotide sequences were analyzed with MEGA X software, and the neighbor-joining phylogenetic tree was constructed with Kimura 2-parameter model and 1,000 bootstrap replications. Following analysis of H9-AIV HA gene by Su et al. (2016), the phylogenetic tree is divided into four genotypic groups (H9.1-H9.4), and the genotypic group H9.4 is subsequently divided into two lineages H9.4.1 and H9.4.2. Interestingly, we noticed that the lineage H9.4.1 is divided into two sub-lineages (which are not previously recorded); H9.4.1.1 sub-lineage contains H9-AIV strains from Egypt (field and vaccine seed strains) and field strains from Libya. H9.4.1.2 sub-lineage contains Asian H9-AIV field strains (from Pakistan, Hong Kong, and Israel) and vaccine seed strains from Saudi Arabia, United Arab Emirates (UAE), and Iran. On the other hand, the sub-lineage H9.4.2 is further divided into six sub-lineages (H9.4.2.1 to H9.4.2.6), and sub-lineage H9.4.2 contains the Chinese strains (Su et al., 2016; Jiang et al., 2012). Our studied strains are aligned in H9.4.1.1 sub-lineage with other Egyptian (field and vaccine seed strains) and Libyan H9-AIV field strains (Figure 4). Our strains are more closely related to the Egyptian H9-AIV field strains from different bird species including ducks (100%), cattle egret (99.6%), pigeon (99.6%), Turtle dove (99.6%), chicken (98.1–99.3%), and quail (97.3%), while the Libyan field strains showed 91.7% identity to our strains. On the other hand, the Asian H9.4.1.2 sub-lineage field strains are somewhat different from our strains with only 87.4–89.3% identity to our isolates. Comparing our strains to local and imported vaccine seed strains used in Egypt, we noticed that the local Egyptian vaccine seed strains are more closely related to our isolates with 96.3 and 98.9% identity to A/chicken/Egypt/S10490/2015 and A/chicken/Egypt/ME543V/2016 vaccine seed strains, respectively. However, the imported vaccine seed strains were somewhat different from our studied strains with 88.7, 88.9, and 89.2% identity to A/chicken/Iran/av1221/1998, A/chicken/Saudi Arabia/CP7/1998, and A/chicken/United Arab Emirates/AG537/99 vaccine seed strains, respectively (Supplementary Table S3). Upon analyzing the deduced amino acid sequences of the H9.4.1 strains (H9.4.1.1 and H9.4.1.2) using BioEdit software, we identified nine conserved amino acid substitutions in the H9.4.1.2 strains compared to the H9.4.1.1 strains (S150A, D183S, T204I, N216D, D218N, I235Q, F264Y, E271G, and G319R) which is shown in Table 4. These substitutions can serve as amino acid signatures for distinguishing between the two sub-lineages (Supplementary Figure S3). The cleavage sites of our studied H9 gene deduced amino acid sequences were compared to other H9.4.1 lineage strains. All aligned H9.4.1 (H9.4.1.1 and H9.4.1.2) strains displayed a very homogeneous HA0 protein cleavage site motif, PARSSR↓GLF except for the Libyan strains, which carried the PSKSSR↓GLF motif (Supplementary Figure S3 and Table 5). This cleavage site motif is characteristic of LPAI as it lacks the multiple basic amino acid residues at the cleavage sites. The receptor binding sites, the right edge Binding pocket (REBP), and the left edge Binding pocket (LEBP) of our strains were also compared to that of local and imported vaccine seed strains used in Egypt. As depicted in Table 5, the receptor binding sites (161 W, 163 T, 166 N, 191H, 197 T, 198A, 201 N, 202 L, and 203Y) were found highly conserved in studied field and vaccine strains except A/chicken/Saudi Arabia/CP7/1998 vaccine seed strain which has two amino acid substitutions (A198E and N201S) and A/chicken/Iran/av1221/1998 vaccine seed strain which has a single amino acid substitution (N166S). Table 5 shows that the right edge Binding pocket (aa143-150) and the left edge Binding pocket (aa 232–237) showed a highly conserved amino acid sequence among Egyptian field strains and local vaccine seed strains while the three imported vaccine seed strains have two amino acid substitutions in the REBP (S145T and S150A) and another two amino acid substitutions in the LEBP (L234Q and I235Q).

**Table 4:** Conserved amino acid substitutions among H9.4.1.2 strains when compared to H9.4.1.1 strains.

Genotype	aa no.								
	150	183	204	216	218	235	264	271	319
H9.4.1.1	S	D	T	N	D	I	F	E	G
H9.4.1.2	A	S	I	D	N	Q	Y	G	R

**Table 5:** Receptor binding site, receptor binding pockets, and cleavage site of H9-AIV.

Site	Right edge binding pocket								Receptor binding site									Left edge binding pocket						Cleavage site							
Amino acid no	143–150								161	163	166	191	197	198	201	202	203	232–237						333–341							
Egyptian field strains	T	Y	S	G	T	S	K	S	W	T	N	H	T	A	N	L	Y	N	G	L	I	G	R	P	A	R	S	S	R	G	L
A/chicken/Egypt/ME543V/2016	.	.	.	.	.	.	.	.	.	.	.	.	.	.	.	.	.	.	.	.	.	.	.	.	.	.	.	.	.	.	.
A/chicken/Egypt/S10490/2015	.	.	.	.	.	.	.	.	.	.	.	.	.	.	.	.	.	.	.	.	.	.	.	.	.	.	.	.	.	.	.
A/chicken/UAE/AG537/99	.	.	T	.	.	.	.	A	.	.	.	.	.	.	.	.	.	.	.	Q	Q	.	.	.	.	.	.	.	.	.	.
A/chicken/Saudi Arabia/CP7/1998	.	.	T	.	.	.	.	A	.	.	.	.	.	E	S	.	.	.	.	Q	Q	.	.	.	.	.	.	.	.	.	.
A/chicken/Iran/av1221/1998	.	.	T	.	.	.	.	A	.	.	S	.	.	.	.	.	.	.	.	Q	Q	.	.	.	.	.	.	.	.	.	.

Comparison of the antigenic sites (Antigenic site I and II) and overlapping site of H9-AIV Egyptian field and vaccine seed strain (local and imported) revealed that the antigenic site I (143 T, 166 N, and 170P) was highly conserved among the Egyptian field strains and local vaccine seed. However, the imported vaccine seed strain A/chicken/Iran/av1221/1998 exhibited a single amino acid substitution at position 166 (N166S). On the other hand, antigenic site II (153D, 201 N, and 234 L) was also highly conserved among the Egyptian field strains and local vaccine seed strains, whereas the imported vaccine seed strains exhibited amino acid substitutions. Remarkably, the strain A/chicken/Saudi Arabia/CP7/1998 displayed a completely distinct antigenic site II (153G, 201S, and 234Q), while the strain A/chicken/Iran/av1221/1998 exhibited two amino acid substitutions (153G and 234Q), along with a single substitution at position 234Q in the strain A/chicken/UAE/AG537/99. The overlapping site (141 N, 197 T, and 206 T) remained highly conserved among all Egyptian field strains and vaccine seed strains (both local and imported), except for the strain A/chicken/Iran/av1221/1998, which displayed the T206N substitution (Table 6). Previous studies reported that certain mutations enhanced H9-AIV binding to both avian and human-like receptors, including D153G/N, Q235M, and R254K, but these were not present in all Egyptian field strains and vaccine seed strains (local and imported), except for the A/chicken/Saudi Arabia/CP7/1998 and A/chicken/Iran/av1221/1998 vaccine strains, which harbored the 153G mutation. Other mutations were identified to enhance H9-AIV binding specifically to human-like receptors, such as Q164R, A168D/N, R180Q, S183N, T213A, D216E, T220I, V224L, Q234 L, and V253I. Among these 10 mutations, only three (168 N, 224 L, and 234 L) were observed in all Egyptian field strains and local vaccine seed strains. An additional mutation (180Q) was recorded in the A/chicken/Egypt/S10490/2015 local vaccine seed strain, while the imported vaccine seed strains had a single mutation (220I), as shown in Table 7. All Egyptian H9-AIV field and local vaccine strains exhibited H191 and L234 amino acid residues at the receptor binding site, indicating that these viruses possessed human influenza virus-like receptor specificity. In contrast, the imported vaccine strains used in Egypt displayed H191 and Q234 at the same site. In addition, as shown in Table 5, the avian receptor-specific amino acid 197 T was present in all Egyptian H9-AIV field and vaccine seed strains, both local and imported.

**Table 6:** Antigenic sites and overlapping site of H9-AIV Egyptian field and vaccine strains.

Site	Antigenic site I			Antigenic site II		Overlapping site			
Amino acid no	143 T	166 N	170P	153D	201 N	**234 L**	141 N	197 T	206 T
Egyptian field strains	.	.	.	.	.	.	.	.	.
A/chicken/Egypt/ME543V/2016	.	.	.	.	.	.	.	.	.
A/chicken/Egypt/S10490/2015	.	.	.	.	.	.	.	.	.
A/chicken/UAE/AG537/99	.	.	.	.	.	Q	.	.	.
A/chicken/Saudi Arabia/CP7/1998	.	.	.	G	S	Q	.	.	.
A/chicken/Iran/av1221/1998	.	S	.	G	.	Q	.	.	N

**Table 7:** Mutations enhancing H9-AIV to bind both avian and human-like receptor or human-like receptor only.

Site	Mutations enhancing H9-AIV to bind												
	Avian and human-like receptor			Human-like receptor									
Amino acid no	D153G/N	Q235M	R254K	Q164R	A168D/N	R180Q	S183N	T213A	D216E	T220I	V224L	Q234 L	V253I
Egyptian field strains	D	I	R	Q	N	R	D	T	N	T	L	L	V
A/chicken/Egypt/ME543V/2016	.	.	.	.	.	.	.	.	.	.	.	.	.
A/chicken/Egypt/S10490/2015	.	.	.	.	.	Q	.	.	.	.	.	.	.
A/chicken/UAE/AG537/99	.	Q	.	.	F	.	S	.	D	.	V	Q	.
A/chicken/Saudi Arabia/CP7/1998	G	Q	.	.	S	.	S	.	D	I	M	Q	.
A/chicken/Iran/av1221/1998	G	Q	.	.	S	.	S	.	D	I	M	Q	.

#### 3.3.4 Histopathological examination

**Figure 5:**
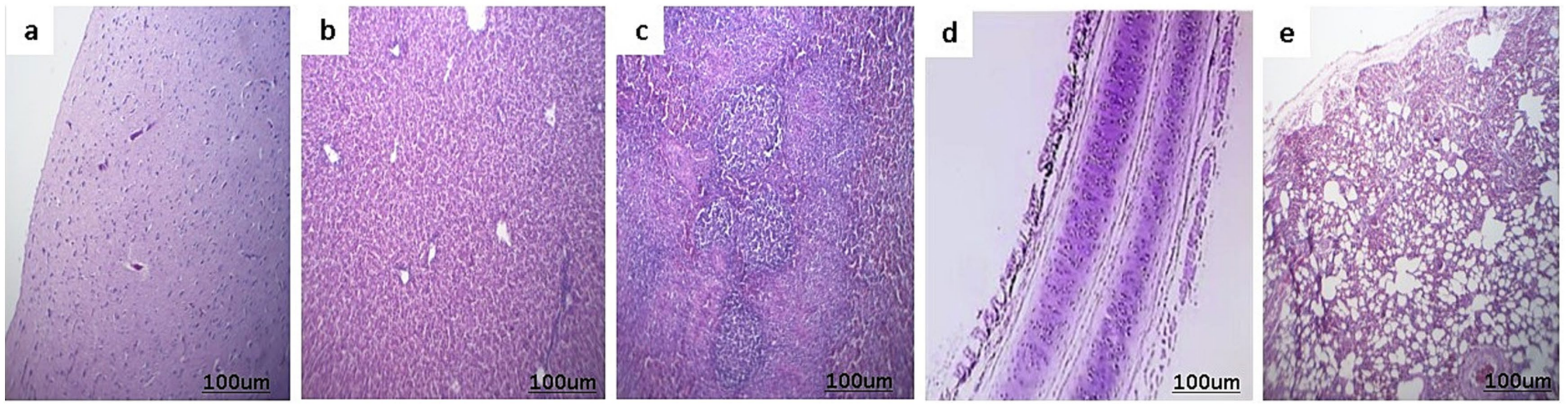
Histopathological picture of control negative ducks stained with H&E. **(a)** Normal brain tissue. **(b)** Normal liver. **(c)** Normal spleen. **(d)** Normal trachea. **(e)** Lungs show normal bronchial epithelium, alveoli, and pulmonary tissue.

The histopathological images of the control negative tissues (brain, liver, spleen, trachea, and lungs) are presented in Figure 5, demonstrating the normal histological architecture of the examined organs. Meanwhile, the histopathological examination of the infected cases revealed the following findings:

##### 3.3.4.1 Histopathological picture of DHAV

**Figure 6:**
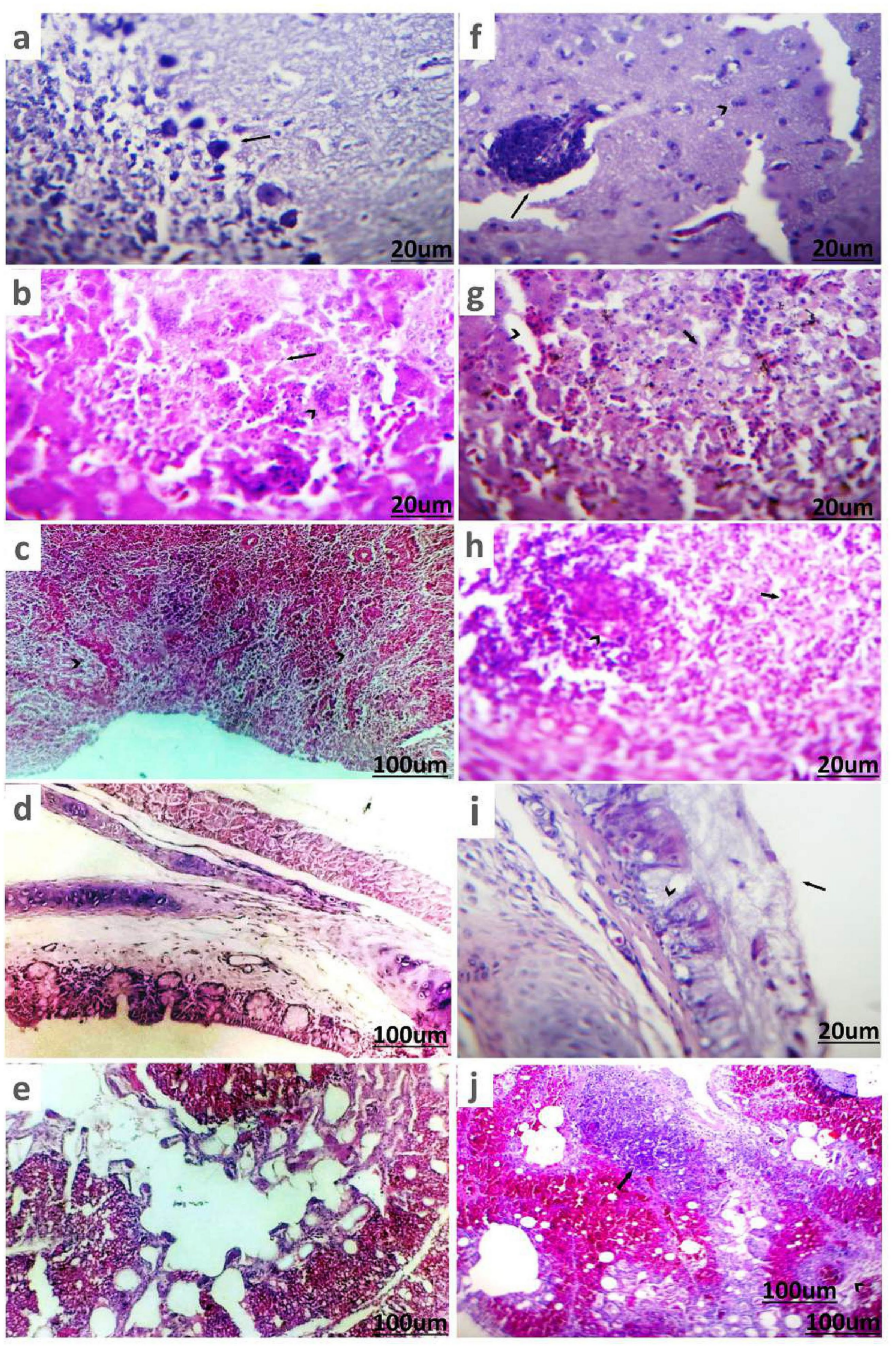
Histopathological picture of ducks infected with DHAV and NDV stained with (H&E). **(a)** Brain tissue of duckling suffering from DHAV shows degenerated Purkinje cells of cerebellum and perineuronal edema (arrow). **(b)** Liver of duckling suffered from DHAV shows area of coagulative necrosis (arrow) infiltrated with macrophages and giant cells (arrowhead). **(c)** Spleen of duckling suffered from DHAV shows focal lymphoid depletion of lymphocytes from white pulp (arrowhead). **(d)** Trachea of duckling suffering from DHAV shows apparently normal mucosa, submucosa, and tracheal ring cartilage. **(e)** Lung of duckling suffered from DHAV showed normal bronchial epithelium, alveoli, and pulmonary tissue. **(f)** Brain tissue of duck suffering from NDV shows perivascular lymphocytic cuffing (arrow) and degenerated neurons (arrowhead) in cerebrum. **(g)** Liver of duck suffered from NDV shows necrotic hepatic cells (arrow) and dilated sinusoids (arrowhead). **(h)** Spleen of duck suffered from NDV shows depletion of white pulps (arrow) and necrotic lymphocytes (arrowhead). **(i)** Trachea of duck suffered from NDV shows thick mucus sticked on mucosa (arrow) with goblet cells metaplasia in glands (arrowhead). **(j)** Lung of duck suffered from NDV shows pneumonic areas (arrow) and severe congestion of blood vessels with perivascular edema (arrowhead).

In DHAV-infected ducks, Purkinje cell neurons of cerebellum underwent degeneration accompanied by perineuronal edema (Figure 6a). The hepatic parenchyma exhibited multiple necrotic areas infiltrated and surrounded by intense inflammatory cells, mainly macrophages with giant cells (Figure 6b). Most of the splenic parenchyma suffered from focal lymphoid depletion of lymphocytes from white pulp (Figure 6c). The trachea showed apparently normal mucosa, submucosa, and tracheal ring cartilage (Figure 6d). The lung showed normal bronchial epithelium, alveoli, and pulmonary tissue (Figure 6e).

##### 3.3.4.2 Histopathological picture of NDV

Examination of the brains of ducks infected with NDV showed that the cerebrum, in particular, had non-purulent encephalitis, which was characterized by perivascular lymphocytic cuffing, focal glial cells aggregates, degenerative neurons, satellitosis, and neuronophagia (Figure 6f). The liver exhibited degenerative or necrotic changes of the hepatic cells, portal leukocytic infiltrates, and hyperemic blood vessels and sinusoids (Figure 6g). The spleen showed depletion of white pulps and necrotic lymphocytes (Figure 6h). The trachea of ducks exhibited adhered mucus on the mucosal surface, along with hyperplastic goblet cells and glands, a few leukocytic infiltrates, and loss of cilia (Figure 6i). The lungs had focal pneumonia manifested by lymphocytic and histiocytic aggregates with thick exudate inside parabronchial air vesicles (Figure 6j).

##### 3.3.4.3 Histopathological picture of H9-AIV

**Figure 7:**
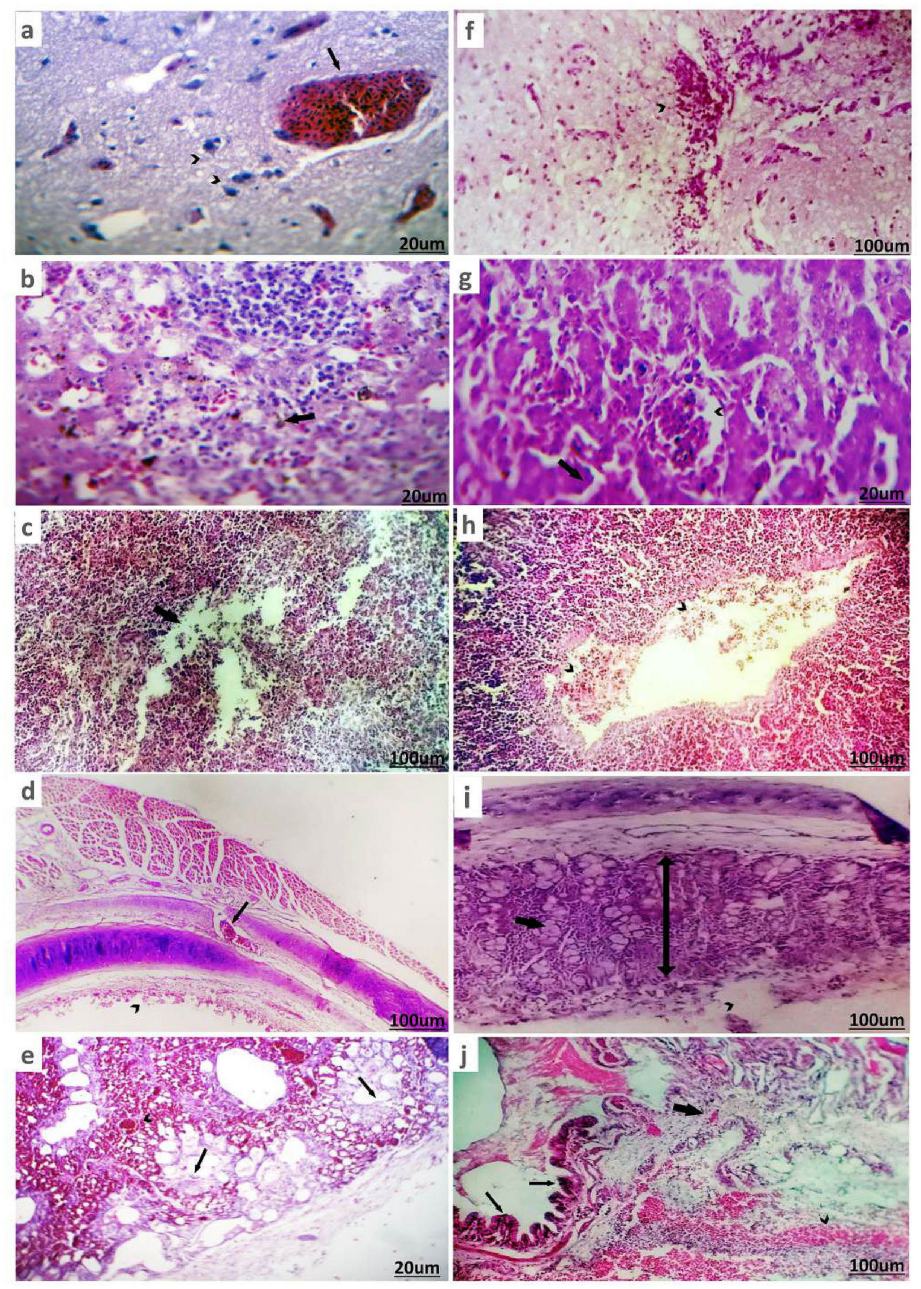
Histopathological pictures of H9-AIV and NDV/ H9-AIV mixed infection of infected ducks stained with (H&E). **(a)** Brain of duck suffered from H9-AIV shows degenerated neurons and perineuronal edema (arrowhead) in addition to severely hyperemic blood vessels (arrow). **(b)** Liver of duck suffered from H9-AIV shows focal necrosis infiltrated and replaced by mononuclear cells (arrow). **(c)** Spleen of duck experienced positivity against H9-AIV showed partial destruction of parenchyma (arrow). **(d)** Trachea of duck suffered from H9-AIV shows submucosal vascular congestion (arrow) and partial mucosal desquamation (arrowhead). **(e)** Lung of duck suffered from H9-AIV showed sero-fibrinous exudate within parabronchi (arrow) and hyperemic blood vessels (arrowhead). **(f)** Brain of duck suffered from NDV/ H9-AIV mixed infection shows focal inflammatory cells infiltration (arrowhead). **(g)** Liver of duck suffered from NDV/ H9-AIV mixed infection shows hemorrhages (arrow) and congested blood vessels (arrowhead). **(h)** Spleen of duck suffered from NDV/ H9-AIV mixed infection shows mild vascular congestion with margination and diapedesis (arrows head). **(i)** Trachea of duck suffered from NDV/ H9-AIV mixed infection partial mucosal destruction (arrowhead) with increase submucosal thickness (double head arrow) and vacuolation of submucosal glands (thick arrow). **(j)** Lung of duck suffered from NDV/H9-AIV mixed infection showed hemorrhagic pneumonia represented in hemorrhage (arrowhead), vascular congestion, inflammatory cell infiltration (thick arrow), and bronchial mucosa hyperplasia (thin arrows).

In ducks infected with H9-AIV, non-suppurative encephalitis was observed, characterized by degenerated or necrotic neurons, satellitosis, and neuronophagia, along with perineuronal edema. In addition, the Virchow-Robin spaces were impacted by extravasated erythrocytes (Figure 7a). The liver had minute necrotic areas infiltrated with mononuclear cells, dilated and hyperemic blood vessels, and sinusoids in addition to hyperplastic Kupffer cells (Figure 7b). Partial destruction of parenchyma was seen in spleen (Figure 7c). Tracheitis is characterized by submucosal vascular congestion (arrow) and partial mucosal desquamation (Figure 7d). Pneumonic areas were scattered primarily around the bronchi, as well as within the parabronchi and air vesicles. Sero-fibrinous exudate, with or without erythrocytes, along with perivascular edema, was commonly observed. In some cases, the bronchi contained intense mucus exudate, with or without erythrocytes (Figure 7e).

### 3.4 Histopathological picture of ducks from mixed infected flocks

The histopathological changes in ducks from NDV/H9-AIV mixed-infected flocks revealed more severe lesions compared to single infections. The brain displayed focal infiltration of inflammatory cells (Figure 7f). The liver showed hemorrhages and congested blood vessels (Figure 7g). The spleen presented mild vascular congestion with margination and diapedesis (Figure 7h). The trachea exhibited partial mucosal destruction, increased submucosal thickness, and vacuolation of submucosal glands (Figure 7i). The lungs showed hemorrhagic pneumonia, characterized by hemorrhage, vascular congestion, inflammatory cell infiltration, and bronchial mucosal hyperplasia (Figure 7j).

## 4 Discussion

Poultry production is regarded as a significant source of income for millions of people globally, with domestic ducks becoming increasingly important in meat and egg production. However, ducks are susceptible to a range of serious viral infections, which can result in considerable economic losses. These losses arise from reduced growth rates, elevated mortality, and increased costs for prevention and control measures, all of which place a significant burden on both the global and Egyptian duck industries (Abd Elfatah et al., 2021; Song et al., 2014). The present study reveals novel molecular and pathological insights into the some of the key viral infections responsible for causing neurological symptoms in ducks in Egypt.

In our study, DHAV was detected in ducklings younger than 21 days, consistent with findings reported by the World Organization for Animal Health (WOAH, 2023). This may be attributed to the hypothesis that the immaturity of their immune systems renders them vulnerable to viral infection and replication. Consequently, the severity of illness in young ducklings infected with DHAV is primarily age-dependent (Abd Elfatah et al., 2021; Song et al., 2014). Furthermore, DHAV caused approximately 55–92% mortality in studied ducks, aligning with findings in previous study (Mansour et al., 2018) noted that duck farms remain at risk for outbreaks linked to DHAV-1, which can exhibit high mortality rates in field conditions ranging from 50 to 95%. A long time ago, the majority strain of DHAV-1 was found in Egyptian duck flocks (Hassan et al., 2020). Nonetheless, recent genetic characterization and phylogenetic investigation of the circulating viruses have identified variants of DHAV-3 that cause notable losses in Egyptian duck flocks (Hassan et al., 2020; Yehia et al., 2021). In our study, both DHAV-1 and DHAV-3 were detected with most tested samples are DHAV-3 (75% of tested samples). DHAV-1 strain was identical to vaccine strain (100% identity). Therefore, this strain should be kept in the vaccine to achieve the best protection rate against DHAV-1, while the DHAV-3 strains exhibited genetic differences from the vaccine strain, showing only 80.3–81.4% identity. These finding which are in concurrence with previous reports (Hassan et al., 2020; Yehia et al., 2021) concluded that the Egyptian DHAV-1-based vaccination is not completely effective in preventing DHAV outbreaks due to the genetic and serological variations between DHAV-1 and DHAV-3 with low cross-protection rate. As a result, this vaccine needs to be modified to be bivalent against both DHAV-1 and DHAV-3, which applied in other countries (Kang et al., 2018; Zou et al., 2016). Despite the 3D gene is confirmed to be a highly conserved gene in DHAV (Hassan et al., 2020), we noticed 14 amino acid substitutions among DHAV genotypes (DHAV-1, DHAV-2, and DHAV-3) which may play a role in genetic variations between different DHAV genotypes. Moreover, the histopathological examination revealed significant focal proliferation of cerebrum glia cells, marked perineural and perivascular edema, and focal demyelination which was previously observed by Hisham et al. (2020). As shown in the histopathological findings, he present study documented a range of hepatic changes in ducks infected with the aforementioned viruses which is in harmony to those documented in previous studies (Fitzgerald et al., 1969; Sheng et al., 2014; Rohaim et al., 2021). Moreover, there were no appreciable variations in the main macroscopic and microscopic lesions in the liver, spleen, and kidneys of the DHAV-infected ducks, aligning with earlier studies (Zhang et al., 2012).

Ducks may serve as both reservoirs and carriers of NDV, as indicated by the presence of pathogenic isolates from ducks and geese. In addition, the number of reported natural NDV cases in ducks has been steadily increasing in recent years, with various NDV strains of differing virulence identified in both clinically healthy and diseased ducks (Zhang et al., 2011; Xu et al., 2017; Higgins, 1971; Cheng et al., 2006; Dai et al., 2014). In this study, NDV was detected in infected ducks with mortality rate of 2–5%, consistently with those previously recorded (Elbestawy et al., 2019). The findings of the current study indicated that the clinical manifestations and PM lesions of the infected ducks were nervous signs (head shaking and lameness or recumbence) and mild signs of enteric disease (greenish diarrhea), with a low mortality rate of 2–5%. These findings are consistent with those published by Bröjer et al. (2013)) and Dai et al. (2014), who suggested that the ND signs in ducks were more passive. According to earlier findings (Liu et al., 2010), localized bleeding and intestinal mucosal necrosis are the predominant signs of the deceased birds. All detected NDV strains in this study are clustered in Genotype VII.1.1 with other Egyptian Genotype VII.1.1 NDV strains from different host species. Recently, Genotype VII.1.1 was the most recorded clade for the Egyptian velogenic NDV and numerous recent outbreaks in Middle East (Abd Elfatah et al., 2021; Song et al., 2014; Miller et al., 2015; Radwan et al., 2013; El-Morshidy et al., 2021). Controlling velogenic NDV infection in ducks seems crucial to prevent interspecies transmission to chickens as it can result in mortality rates of up to 100% in susceptible, unvaccinated chickens (WOAH, 2021).

Taken into account, the virulence of NDV is mainly dependent on the F0 glycoprotein precursor amino acid sequence motif which cleaves into F1 and F2 during NDV replication (Choi et al., 2010; Bello et al., 2018; Awad et al., 2020). This cleavage allows the virus to spread systemically through its detection by widely distributed host proteases (Dortmans et al., 2010). In our study, all studied strains are confirmed to be velogenic NDV strains through the presence of four basic amino acids cleavage site (112-RRQKR-116) with phenylalanine (F) at residue 117 which are all characteristic for velogenic NDV strains as previously confirmed in various previous studies (El-Morshidy et al., 2021; Panda et al., 2004; de Leeuw et al., 2005). BioEdit software analysis of deduced amino acid sequences revealed 17 amino acid substitutions in Egyptian velogenic GVII.1.1 strains when compared to lentogenic GII vaccine strains which can be used as a signature for differentiation between velogenic GVII.1.1 and lentogenic GII NDV strains. These results are strengthened by a previous study (Mouhamed et al., 2020) recorded amino acid signature for the Egyptian velogenic GVII.1.1 NDV strains, including V52, R71, E82, G104, S107, K115, F117, and S124. Furthermore, the presence of amino acid V52 and S176 was previously considered as a specific signature to Genotype VII.1.1 (de Almeida et al., 2013). The histopathological findings associated with NDV cases in brain in the present study included encephalitis represented by vasculitis, congestion, and perivascular edema, which are in accordance with a previous reports (Ecco et al., 2011; Etriwati et al., 2017). Although NDV primarily targets lymphoid tissues, particularly in the spleen and other lymphoid organs, the viral protein was also distinctly detected in the renal epithelium, as well as in the respiratory epithelium of the trachea, lungs, and air sacs (Susta et al., 2014). When the lungs were obstructed, the liver showed severe congestion, which is also supported by previous reports (Anis et al., 2013). A previous research (El-Morshidy et al., 2021) linked NDV to pneumonia, lymphoid depletion, neurological lesions, and epithelial cell necrosis in ducks. Moreover, a previous research (Ecco et al., 2011) reported lymphoid organs lesions consisted of severe necrosis, marked lymphocyte depletion, infiltration of numerous macrophages, and moderate heterophilic infiltrate. Moreover, the reported lesions in lung in some cases could be a result of circulatory disturbance caused by viremia and bacterial secondary infection (Lopez, 2012).

It is noteworthy to mention that H9-AIV is considered the most prevalent AIV in poultry worldwide and has been reported as endemic in Egyptian poultry populations since 2011, causing mild-to-severe respiratory symptoms (Peacock et al., 2019; Abdel-Moneim et al., 2012). Although H9-AIV is classified as a low pathogenic virus, it can lead to substantial financial losses in the poultry sector, particularly due to its potential to increase disease severity when co-infections with other viruses occur (Sun and Liu, 2015; Pusch and Suarez, 2018). The LPAI H9 virus unstable genome mutates continuously due to antigenic drift in the HA gene. Among others, H9-AIV is key participants in these shifts, often donating internal genes to other AIVs during co-infection. Effective control requires effective vaccination as part of an integrated disease control program. Inactivated vaccines in Egypt face challenges due to HA homology and antigen content (Kandeil et al., 2017; Shelkamy et al., 2022). One of the thorniest problems with the H9-AIV is its transmission from ducks to chickens, with its shedding for a long time, which caused circulation of the virus among ducks and chicken (Joseph et al., 2017). Consequently, it seems very critical to maintain ongoing surveillance of LPAIVs like H9-AIV in waterfowl like ducks to successfully prevent and control the infections (Zhao et al., 2016). The present study described H9-AIV virus detection in ducks suffering from respiratory manifestation and greenish diarrhea and a somewhat higher death rate 15–20%, aligning with those previously reported (Pillai et al., 2010). The phylogenetic analysis revealed that our studied strains are aligned in H9.4.1.1 sub-lineage with other Egyptian field strains from different bird species, including ducks (100%), cattle egret (99.6%), pigeon (99.6%), Turtle dove (99.6%), chicken (98.1–99.3%), and quail (97.3%). This high identity percent between H9-AIV strains from different species proposed the inter-species circulation of the virus (Joseph et al., 2017). Comparing our strains to local and imported vaccine seed strains used in Egypt, the local Egyptian vaccine seed strains were found more closely related to our isolates with 96.3 and 98.9% identity. However, the imported vaccine seed strains were somewhat different from our studied strains with 88.7–89.2% identity. Clearly, it is recommended to use the local H9-AIV vaccines for more protection of duck flocks. According to a previous study (Su et al., 2016), the phylogenetic tree is categorized into four genotypic groups (H9.1 to H9.4), with the H9.4 group further divided into two lineages: H9.4.1 and H9.4.2. Notably, we identified two previously unrecorded sub-lineages within H9.4.1: the H9.4.1.1 sub-lineage, which includes Egyptian H9-AIV strains (both field and vaccine seed strains) as well as field strains from Libya; and the H9.4.1.2 sub-lineage, which comprises Asian H9-AIV field strains from Pakistan and Hong Kong, along with vaccine seed strains from Saudi Arabia, the United Arab Emirates (UAE), and Iran. Using BioEdit software, we identified nine conserved amino acid substitutions (S150A, D183S, T204I, N216D, D218N, I235Q, F264Y, E271G, and G319R) in H9.4.1.2 strains compared to H9.4.1.1. These substitutions serve as distinctive signatures for differentiating the two sub-lineages, and, to our knowledge, these findings have not been previously reported.

The cleavage site motif of LPAI is characterized by lacking the multiple basic amino acid residues at the cleavage sites. All our studied strains displayed the characteristic HA0 protein cleavage site motif of LPAI (PARSSR↓GLF) which was recorded in various previous reports (Su et al., 2016; Kandeil et al., 2017; Pillai et al., 2010). The receptor binding sites, REBP, and the LEBP of our strains were also compared to that of local and imported vaccine seed strains used in Egypt. The receptor binding sites (161 W, 163 T, 166 N, 191H, 197 T, 198A, 201 N, 202 L, and 203Y) identified by in a previous study (Kandeil et al., 2017), along with the REBP (aa143-150) and LEBP (aa232-237), exhibited a highly conserved amino acid sequence among Egyptian field strains and local vaccine seed strains, with some amino acid substitutions noted in imported vaccine seed strains. These findings, previously recorded in a previous study, underscored the necessity of using local vaccines to enhance protection for susceptible birds (Shelkamy et al., 2022).

The antigenic sites (Antigenic site I and II) and overlapping site of H9-AIV were previously studied (Kandeil et al., 2017). The Egyptian field and vaccine seed strain (local and imported) revealed that the antigenic site I (143 T, 166 N, and 170P) and the overlapping site (141 N, 197 T, and 206 T) are highly conserved in the Egyptian field strains and local vaccine seed strains with some amino acid substitutions in imported vaccine seed strain A/chicken/Iran/av1221/1998 which are in concurrence with previous investigations (Kandeil et al., 2017; Shelkamy et al., 2022). On the other hand, the antigenic site II (153D, 201 N, and 234 L) was also highly conserved in the Egyptian field strains and local vaccine seed strains with some amino acid substitutions in imported vaccine seed strains including A/chicken/Saudi Arabia/CP7/1998 which has a totally different antigenic site II, but a previous study (Shelkamy et al., 2022) reported no mutations in antigenic site II. Another previous study (Jiang et al., 2012) confirmed that the lineages H9.4.1 and H9.4.2 are of higher risk to the general public’s health due to the presence of 234 L. The amino acid 234 L at the receptor binding site of H9-AIV has human influenza virus-like specificity due to its affinity to bind *α* 2,6-linked sialic acid which is the main receptor in human respiratory tract, while the α2,3 receptors were mainly distributed in avian species (Jiang et al., 2012; Wan et al., 2008; Sorrell et al., 2009). The amino acid 234 L was present in all studied Egyptian H9-AIV with higher binding affinity to human-like α 2,6 sialic acid receptors than avian α 2,6 sialic acid receptors as previously reported (Kandeil et al., 2017). Interestingly, a previous study (Liu et al., 2020) reported that other mutations (D153G/N, Q235M, and R254K) are known to enhance H9-AIV binding to both avian and human-like receptors but they were not present in all Egyptian field strains and local vaccine seed. Other mutations were reported to enhance H9-AIV binding to human-like receptors only as Q164R, A168D/N, R180Q, S183N, T213A, D216E, T220I, V224L, Q234 L, and V253I (Liu et al., 2020); from these 10 mutations, only three mutations (168 N, 224 L, and 234 L) were observed in all Egyptian field strains and local vaccine seed strains, and an additional mutation (180Q) is recorded in A/chicken/Egypt/S10490/2015 local vaccine seed strain increasing the ability of the studied virus to infect humans (Liu et al., 2020). All the aligned Egyptian H9-AIV field and local vaccine strains have H191, 197 T, and L234 amino acid residues at the receptor binding site, indicating that these viruses had the characteristic of human influenza virus-like receptor specificity. These results are consistent with previous studies (Sorrell et al., 2009; Fusaro et al., 2011) which concluded that the presence of these amino acids at the receptor binding site is essential for the transmission of respiratory droplet containing a reassorting virus with H9-AIV in a human H3N2 backbone. A previous research (Cox et al., 2016) illustrated that certain strains of H9-AIV that have occasionally caused sporadic zoonotic infections have the potential to cause a pandemic if additional mutations allow for persistent human-to-human transmission. Based on previous studies (Jiang et al., 2012; Saito et al., 2001; Butt et al., 2005), H9-AIV is considered one of the most likely candidates for triggering a new influenza pandemic in humans. Therefore, attention should be paid to the molecular nature of such an important virus to avoid its zoonotic risk. As previously reported (Zhu et al., 2018), histopathological analysis showed that the respiratory system was severely impacted by H9-AIV. The current study revealed the presence of inflammatory lesions in the trachea, bronchus, and parabronchus, accompanied by focal infiltration of inflammatory cells in the bronchial wall. Notably, lymphocytes and fibrin exudate were the primary contributors to this inflammatory response in the parabronchus, accompanied by mild hemorrhaging. While a previous study (Wang et al., 2014) reported that lesions were limited to the trachea and lungs, characterized by congestion and mild mononuclear cell infiltration, the same study noted that other organs, including the gastrointestinal tract, which is considered the primary sites of LPAIV replication in ducks, did not exhibit significant microscopic lesions. In addition, mixed viral infections were associated with higher mortality rates, more pronounced clinical signs, and severe post-mortem lesions, as previously confirmed (Mansour et al., 2018). Marked reduction of lymphocytes in the spleen, depletion of lymphoid follicles, and necrosis was observed by Eladl et al. (2020). Another study (Awadin et al., 2018) reported that liver showed congestion of portal veins with periportal aggregation of leukocytes mainly lymphocytes and macrophages. This study is limited by the pooling of tissues from each farm, a method influenced by logistical and funding constraints. While this approach restricts individual-level analysis of mixed infections, the findings provide valuable molecular and pathological insights into key neurotropic viral infections in Egyptian ducks. Our conclusion on the NDV analysis remains preliminary as the BioEdit analysis includes only two GII strains, and therefore, further investigation with a larger dataset is needed for a more comprehensive understanding. Another key limitation of this study is the use of the DHAV-3D gene instead of VP1 for phylogenetic analysis, which, while providing a stable molecular marker, may overlook important antigenic variations. In addition, the classification of NDV was based on a partial F gene sequence rather than the full-length gene, which may limit resolution in detecting recombination events or novel mutations. Future studies incorporating VP1 for DHAV and full-length F gene sequencing for NDV are recommended to enhance the accuracy of phylogenetic and evolutionary analyses.

## 5 Conclusion

The present study concluded that both single and co-infections were identified, with co-infections leading to higher mortality rates in the affected ducks. Consequently, further research involving a larger duck population is recommended to better understand the implications of these findings. Regarding DHAV, most tested samples contain DHAV-3 genotype which is genetically divergent from the DHAV-1 vaccine used in Egypt which should be updated to contain both DHAV-1 and DHAV-3. All studied NDV strains were identified as velogenic, highlighting the importance of controlling these infections to prevent transmission to chickens, which can lead to mortality rates of up to 100% in susceptible populations. It is advisable to utilize local Egyptian H9-AIV vaccine seed strains, as they exhibit a closer genetic relationship to our isolates compared to imported vaccines. Furthermore, all Egyptian H9-AIV strains demonstrated receptor specificity akin to that of human influenza viruses, thereby increasing the zoonotic risk associated with this virus. Continuous surveillance of NDV in ducks is an important issue as all studied strains circulating in Egyptian ducks are velogenic NDV strains which can be a source of chicken infection causing up to 1–00% of infected chickens causing more economic loses.

Institutional Review Board Statement and Ethics approval: The study, including duck handling, management practices, and adherence to biosecurity and biosafety protocols, was fully compliant with the ‘Guidelines for the Care and Use of Laboratory Animals.’ All procedures were approved by the Research Ethical Committee of the Faculty of Veterinary Medicine, Mansoura University (MU-ACUC; Approval Code: VM.R.24.01.140).

## Data Availability

The original contributions presented in the study are included in the article/Supplementary material, further inquiries can be directed to the corresponding author.

## Footnotes

^1^ http://WWW.ncbi.nlm.nih.gov/Genbank
