# Supplementary material for: A comprehensive pathological and molecular investigation of viral co-infections in ducks in Egypt
Source: Front Microbiol. 2025 May 8;16:1522669. doi: 10.3389/fmicb.2025.1522669 (PMC12097280; doi:10.3389/fmicb.2025.1522669)
Supplement: Supplementary file 1 [file Data_Sheet_1.ZIP › Supplementary tables S1-1_R2 22.03.2025/Supplementary table S1-1_R1 02.03.2025.docx]

Table S1: details of studied samples including sample no, collection date, breed of studied birds, age, mortality rate, clinical signs, PM lesions, detected virus and acession no of sequenced strains.

| Sample no | Collection date | Duck breed | Age  (days) | Mortality rate | Clinical signs | PM lesions | Detected virus (es) | Accession no of sequenced strains |
| --- | --- | --- | --- | --- | --- | --- | --- | --- |
| 1 | Oct.-2022 | Mullard | 14 | 70% | depression, anorexia, pyrexia, feather discoloration, lassitude, wing drooping, ataxia, paddle spasmodically | reddish discoloration of liver, enlarged and mottled spleen | DHAV-1/NDV | OQ376755 (DHAV-1)  OQ376751 (NDV) |
| 2 | Oct.-2022 | Pekin | 30 | 4% | anorexia, watery, greenish-white diarrhea, emaciation, lay on their sides and exhibited a swimming motion with both legs and some birds began to show other loss of balance | Brain edema, congested tracheal mucosa, and extensive small intestine hemorrhages were found. | NDV |  |
| 3 | Nov.-2022 | Baladi | 10 | 90% | depression, anorexia, pyrexia, feather discoloration, lassitude, wing drooping, ataxia, loss of balance, paddling spasmodically | reddish discoloration of liver, enlarged and mottled spleen | DHAV-3 | OQ376756 |
| 4 | Nov.-2022 | Baladi | 12 | 60% | depression, anorexia, fever, feather discoloration, lassitude, wing drooping, ataxia, diarrhea, emaciation, swimming motion, neurological signs, gasping, and nasal discharge. | Mottled liver, enlarged spleen, swollen kidneys, hemorrhages, brain congestion, congested trachea, pneumonia, air sacculitis, and pancreatitis. | NDV/H9-AIV |  |
| 5 | Sep-2022 | Mullard | 7 | 70% | depression, anorexia, pyrexia, feather discoloration, lassitude, wing drooping, ataxia, diarrhea, emaciation, swimming motion, loss of balance, and gasping. | mottled liver, enlarged spleen, swollen kidney, hemorrhages, meningitis, brain congestion, tracheal congestion, severe hemorrhages, tracheitis, pneumonia, air sacculitis, splenomegaly, pancreatitis, enteritis, nephritis. | NDV/H9-AIV | OQ376752 (NDV)  OQ346183 /A/Duck/Egypt/22-1/2022 (H9-AIV) |
| 6 | Sep-2022 | Pekin | 90 | 20% | depression, decreased feed consumption, gasping, nasal discharge and white-greenish diarrhea | tracheitis, pneumonia, air sacculitis, splenomegaly, pancreatitis, enteritis, and nephritis | H9-AIV |  |
| 7 | Nov. -2022 | Baladi | 15 | 85% | depression, anorexia, pyrexia, feather discoloration, lassitude, wing drooping, ataxia, loss of balance, paddle spasmodically | mottling liver, enlarged spleen with mottling and swollen kidney | DHAV/ H9-AIV |  |
| 8 | Nov.-2022 | Baladi | 50 | 5% | anorexia, watery greenish-white diarrhea, paresis, emaciated, unable to rise, lack of coordination, circling, muscular tremors | brain hemorrhages, diffuse brain edema, , congested tracheal mucosa, extensive hemorrhages in the mucosa of small intestine | NDV |  |
| 9 | Oct-2022 | Mullard | 10 | 67% | Rise, lay on sides, lose balance, paddle spasmodically, lassitude, drooping wings, ataxia. | mottling liver, enlarged spleen with mottling and swollen kidney | DHAV |  |
| 10 | Oct-2022 | Mullard | 14 | 60% | emaciated, unable to rise, lay on their sides, lose balance, paddle spasmodically | reddish discoloration of liver, enlarged and mottled spleen | DHAV |  |
| 11 | Sep-2022 | Pekin | 16 | 55% | anorexia, and watery, greenish-white diarrhea, loss of balance, opisthotonus, and torticollis | enlarged liver covered with hemorrhagic foci, enlarged spleen and mottled, enlarged kidneys with congested blood renal vessels | DHAV |  |
| 12 | Nov. -2022 | Baladi | 50 | 15% | depression, decreased feed consumption and respiratory signs, sneezing and coughing, rales, nasal discharge, white-greenish diarrhea | tracheitis, pneumonia, air sacculitis, splenomegaly, pancreatitis, enteritis, and nephritis | H9-AIV | OQ346185/A/Duck/Egypt/22-3/2022 (H9-AIV) |
| 13 | Sep-2022 | Mullard | 85 | 40% | Weak and unable to rise, they lay on their sides with a swimming motion of their legs, twisting of the head and neck, lack of coordination, circling, and muscle tremors. | atrophied, friable spleen, Congestion, and necrosis of pancreas. Severe hemorrhages of thymus and bursa, petechiae Hemorrhages in the mucosa of the proventriculus | NDV/H9-AIV | OQ376753 (NDV)  OQ346184/A/Duck/Egypt/22-2/2022 (H9-AIV) |
| 14 | Oct. -2022 | Mullard | 12 | 93% | anorexia, and watery, greenish-white diarrhea, lay on their sides and exhibited a swimming motion with both legs, | Severe hemorrhages of thymus and bursa, extensive hemorrhages in the mucosa of small intestine | DHAV/NDV |  |
| 15 | Sep-2022 | Mullard | 8 | 88% | depression, anorexia, pyrexia, feather discoloration, ataxia, emaciation, watery diarrhea, decreased feed consumption, and respiratory signs. | mottling liver, enlarged spleen, swollen kidney, hemorrhages, brain congestion, tracheitis, pneumonia, and pancreatitis.. | DHAV-3/H9-AIV | OQ376757 (DHAV-3)  OQ346186/A/Duck/Egypt/22-4/2022 (H9-AIV) |
| 16 | Nov. -2022 | Pekin | 14 | 92% | emaciated, unable to rise, and they lay on their sides, lassitude, wing drooping, ataxia, loss of balance, paddle spasmodically | Mottled liver, enlarged spleen, swollen kidney, spotted hemorrhages in endocardium and epicardium, edema, and hyperemia of meninges. | DHAV-3 | OQ376758 |
| 17 | Oct. -2022 | Pekin | 30 | 2% | lack of muscular coordination, circling, and muscular tremors anorexia, greenish-white diarrhea | congestion or hemorrhages in the brain diffuse brain edema were, found, congested tracheal mucosa, extensive hemorrhages in the mucosa of small intestine | NDV | OQ376754 |
| 18 | Sep-2022 | Baladi | 60 | 40% | depression, decreased feed consumption and respiratory signs, sneezing and coughing, rales, nasal discharge, white-greenish diarrhea | tracheitis, pneumonia, air sacculitis, splenomegaly, pancreatitis, enteritis, and nephritis | NDV/H9-AIV |  |
| 19 | Nov.-2022 | Baladi | 90 | 3% | Anorexia, paresis, emaciation, inability to rise, lying on sides, and exhibiting swimming motions with legs, along with twisting of head and neck. | Brain edema, tracheal mucosa congestion, and extensive small intestine hemorrhages were observed. | NDV |  |
| 20 | Oct. -2022 | Baladi | 12 | 80% | emaciated, unable to rise, and they lay on their sides, lassitude, wing drooping, ataxia, loss of balance, paddle spasmodically | mottling liver, enlarged spleen with mottling and swollen kidney | DHAV-3 | OQ376759 |
