# Supplementary material for: A comprehensive pathological and molecular investigation of viral co-infections in ducks in Egypt
Source: Front Microbiol. 2025 May 8;16:1522669. doi: 10.3389/fmicb.2025.1522669 (PMC12097280; doi:10.3389/fmicb.2025.1522669)
Supplement: Supplementary file 2 [file Data_Sheet_2.ZIP › Supplementary figures R_02.03.2025/Supplementary figures R_02.03.2025.docx]

**
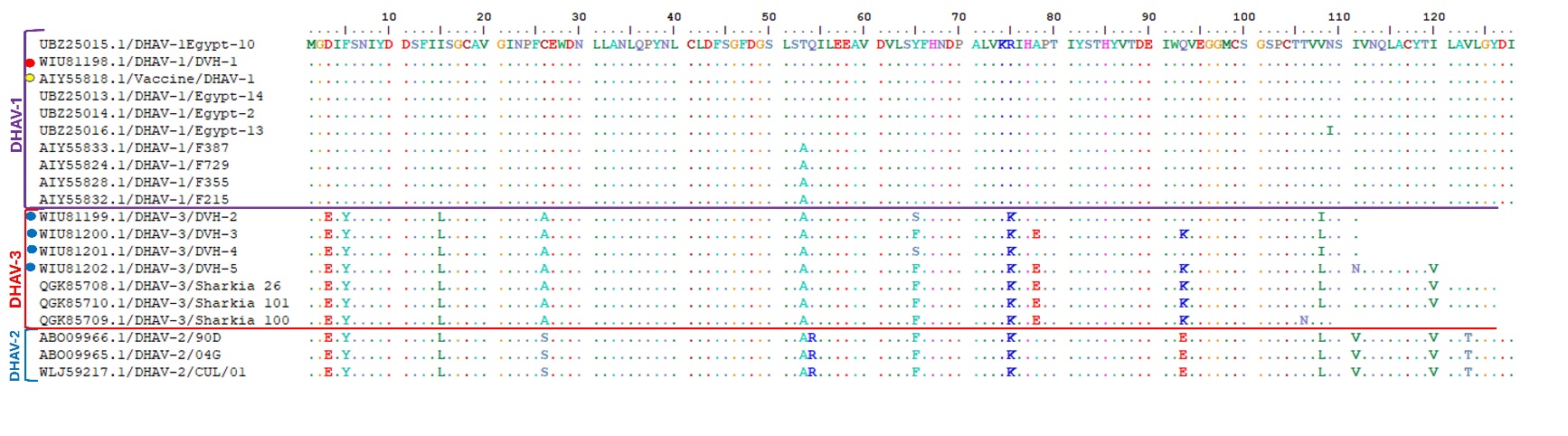
Figure S1.** BioEdit alignment of DHAV- 3D gene deduced amino acid sequences of Egyptian DHAV-1, and DHAV-3 isolates together with DHAV-2 strains from GenBank. Alignment revealed amino acid substitutions among DHAV-1, DHAV-2 and DHAV-3. Red and Blue circles: our studied strains and the yellow circle: Egyptian vaccine strain. The strains WIU81199, WIU81201 and WIU81202 represent individual DHAV-3 infection while the strain WIU81198 represents DHAV-1/NDV mixed infection and the strain WIU81201 represents DHAV-3/H9-AIV mixed infection.


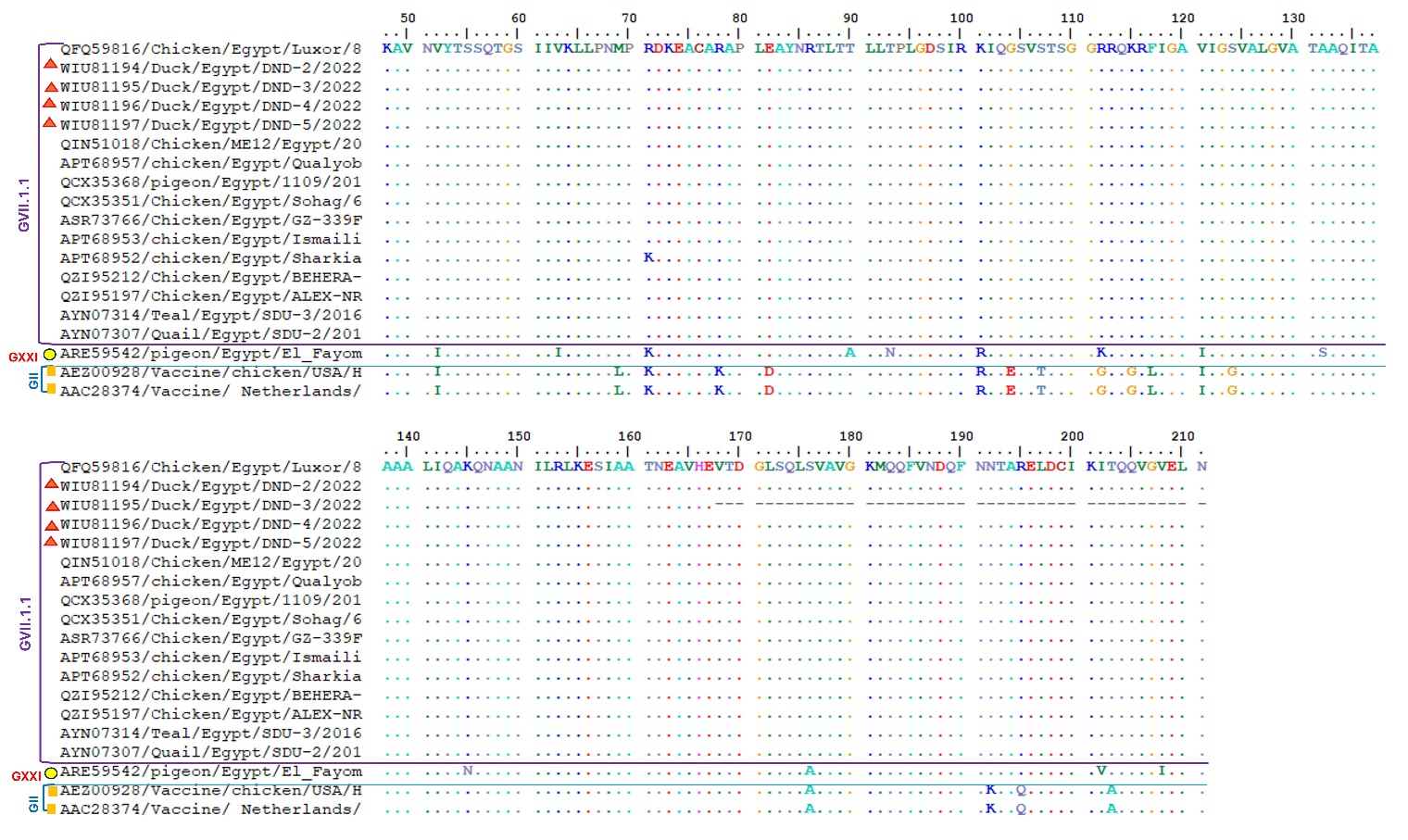
**Figure S2.** BioEdit alignment of NDV- F gene deduced amino acid sequences of Egyptian GVII.1.1, and GXXI isolates together with GII vaccine strains from GenBank. The amino acid numbering was done according to Luxor/8 reference GVII.1.1 Egyptian strain. Alignment revealed amino acid substitutions among GVII.1.1, GXXI, and GII. Red triangles: our studied strains, the yellow circle: Egyptian GXXI strain and the orange squares: GII vaccine strains. The strain WIU81197 represents individual NDV infection while the strains WIU81195 and WIU81196 represents NDV/H9-AIV mixed infection and the strain WIU81194 represents DHAV-1/NDV mixed infection.


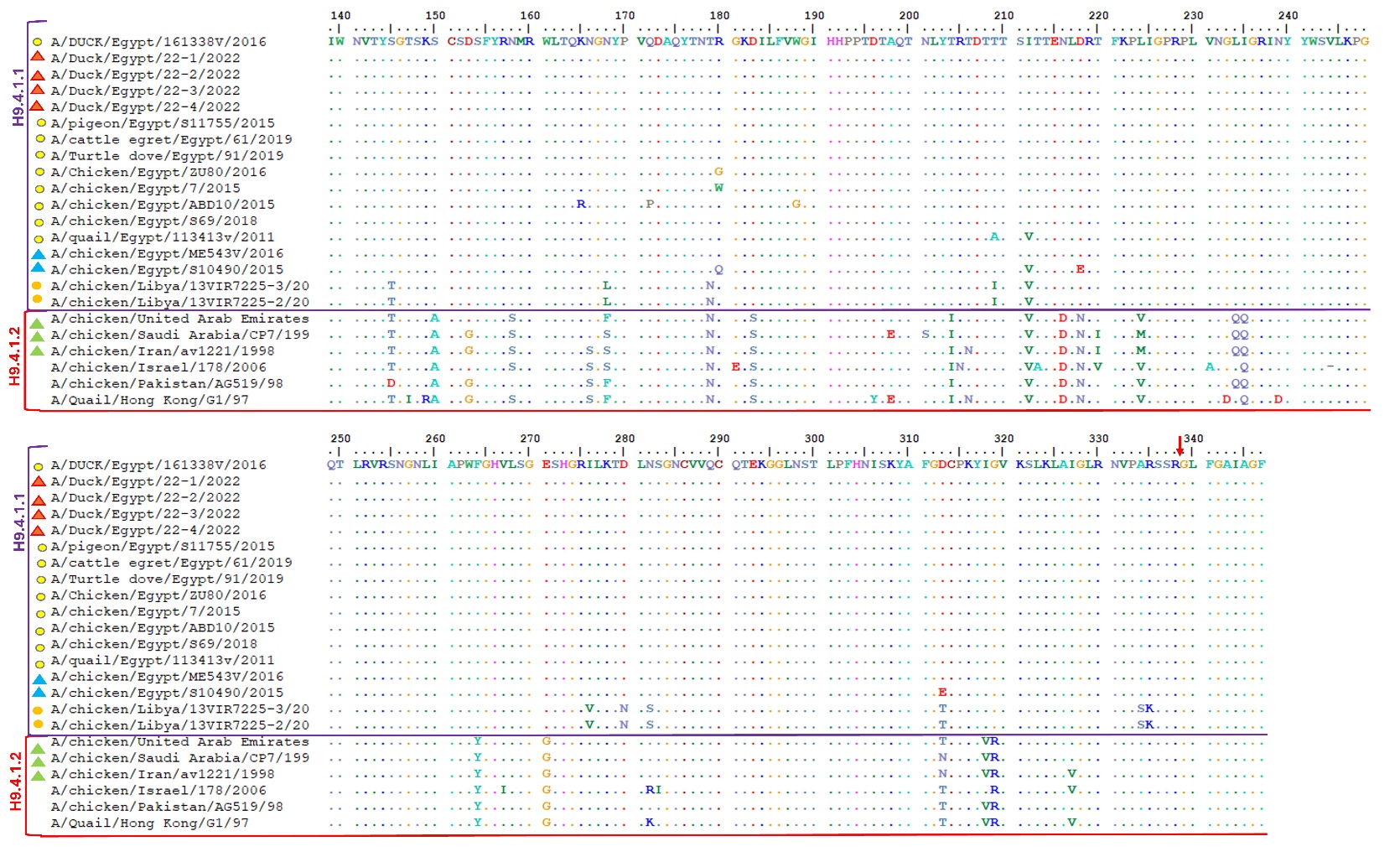
**Figure S3.** BioEdit alignment of H9-AIV HA gene deduced amino acid sequences of Egyptian H9.4.1.1 field strains and local vaccine strains together with H9.4.1.2 Asian field and vaccine strains from GenBank. The amino acid numbering is according H9 numbering of Gao et al., 2021. Alignment revealed several amino acid substitutions among H9.4.1.1 and H9.4.1.2 strains. Red triangles: our studied strains, the yellow circle: Egyptian field strain, blue triangles: local Egyptian vaccine seed strains, orange circle: Libyan strains, green triangles: imported vaccine strains used in Egypt and the red arrow represents the HA0 cleavage site. The strain A/Duck/Egypt/22-3/2022 represents individual H9-AIV infection while the strains A/Duck/Egypt/22-1/2022 and A/Duck/Egypt/22-2/2022 represent NDV/H9-AIV mixed infection and the strain A/Duck/Egypt/22-4/2022 represents DHAV-3/H9-AIV mixed infection.
